# Unraveling astrocyte behavior in the space brain: Radiation response of primary astrocytes

**DOI:** 10.3389/fpubh.2023.1063250

**Published:** 2023-04-06

**Authors:** Marie Denise Roggan, Jessica Kronenberg, Esther Wollert, Sven Hoffmann, Hasan Nisar, Bikash Konda, Sebastian Diegeler, Christian Liemersdorf, Christine E. Hellweg

**Affiliations:** ^1^Department of Radiation Biology, Institute of Aerospace Medicine, German Aerospace Center (DLR), Cologne, Germany; ^2^Microgravity User Support Center (MUSC), German Aerospace Center (DLR), Cologne, Germany; ^3^Department of Gravitational Biology, Institute of Aerospace Medicine, German Aerospace Center (DLR), Cologne, Germany; ^4^Department of Medical Sciences, Pakistan Institute of Engineering and Applied Sciences (PIEAS), Islamabad, Pakistan; ^5^Department of Radiation Oncology, UT Southwestern Medical Center, Dallas, TX, United States

**Keywords:** astrocytes, X-rays, heavy ions, simulated microgravity, DNA double strand breaks, cytokines, cell cycle, astrocyte reactivity

## Abstract

**Introduction:**

Exposure to space conditions during crewed long-term exploration missions can cause several health risks for astronauts. Space radiation, isolation and microgravity are major limiting factors. The role of astrocytes in cognitive disturbances by space radiation is unknown. Astrocytes' response toward low linear energy transfer (LET) X-rays and high-LET carbon (^12^C) and iron (^56^Fe) ions was compared to reveal possible effects of space-relevant high-LET radiation. Since astronauts are exposed to ionizing radiation and microgravity during space missions, the effect of simulated microgravity on DNA damage induction and repair was investigated.

**Methods:**

Primary murine cortical astrocytes were irradiated with different doses of X-rays, ^12^C and ^56^Fe ions at the heavy ion accelerator GSI. DNA damage and repair (γH2AX, 53BP1), cell proliferation (Ki-67), astrocytes' reactivity (GFAP) and NF-κB pathway activation (p65) were analyzed by immunofluorescence microscopy. Cell cycle progression was investigated by flow cytometry of DNA content. Gene expression changes after exposure to X- rays were investigated by mRNA-sequencing. RT-qPCR for several genes of interest was performed with RNA from X-rays- and heavy-ion-irradiated astrocytes: *Cdkn1a, Cdkn2a, Gfap, Tnf*, *Il1*β, *Il6*, and *Tgf*β*1*. Levels of the pro inflammatory cytokine IL-6 were determined using ELISA. DNA damage response was investigated after exposure to X-rays followed by incubation on a 2D clinostat to simulate the conditions of microgravity.

**Results:**

Astrocytes showed distinct responses toward the three different radiation qualities. Induction of radiation-induced DNA double strand breaks (DSBs) and the respective repair was dose-, LET- and time-dependent. Simulated microgravity had no significant influence on DNA DSB repair. Proliferation and cell cycle progression was not affected by radiation qualities examined in this study. Astrocytes expressed IL-6 and GFAP with constitutive NF-κB activity independent of radiation exposure. mRNA sequencing of X-irradiated astrocytes revealed downregulation of 66 genes involved in DNA damage response and repair, mitosis, proliferation and cell cycle regulation.

**Discussion:**

In conclusion, primary murine astrocytes are DNA repair proficient irrespective of radiation quality. Only minor gene expression changes were observed after X-ray exposure and reactivity was not induced. Co-culture of astrocytes with microglial cells, brain organoids or organotypic brain slice culture experiments might reveal whether astrocytes show a more pronounced radiation response in more complex network architectures in the presence of other neuronal cell types.

## 1. Introduction

Long-term space travel and planetary exploration, including missions to Moon and Mars, are the next challenging steps for crewed space missions. During these missions, exposure to space radiation might be detrimental to astronaut health as a risk factor for the development of cancer and non-cancer effects (1, 2). There are two major sources for the space radiation environment in space: (1) Galactic cosmic rays (GCR) originating beyond the solar system [energetic protons, helium nuclei and heavy ions, also called high charge and energy (HZE) nuclei] and (2) Solar energetic particles (SEP) continuously emitted by the Sun as low energetic solar wind or during coronal mass ejections as solar particle events (SPE). While GCR are composed of 98 % baryons (87 % protons, 12 % helium ions, ~1 % heavy ions) and 2 % electrons and positrons (3), SPE are composed of 89 % protons, 10 % helium ions and 1 % heavy ions. The probability of their occurrence rises during the solar maximum of the ~11-year solar cycle. As GCR cannot be completely shielded during free space voyage, a chronic low-dose rate GCR exposure of astronauts accumulates to considerable doses during a 3-year-Mars mission (~1 Sv) (4–6). Furthermore, SPE bear the risk of acute exposure to high dose rates in situations of insufficient shielding.

With this composition, space radiation differs strongly from well-characterized ionizing radiation qualities on Earth such as alpha-, beta- and gamma radiation or X-rays. High complexity of the space radiation field makes assessment of its biological effects quite difficult. GCR simulation composed of protons, helium nuclei and selected heavy ions became only recently available at the NASA Space Radiation Laboratory (NSRL) at Brookhaven National Laboratories (BNL) (7). Mostly, experiments with single mono-energetic ions are performed to assess their biological effectiveness in comparison to well-known radiation qualities such as X-rays or gamma rays. Here, the linear energy transfer (LET) of radiation is frequently used to describe ionization density and correlated biological effects. While X-rays or γ-rays are considered low-LET radiation, HZE particles of GCR are high-LET radiation.

Due to its limited repair capacity, space radiation effects on the central nervous system (CNS) are of high interest. Decreased CNS performance, but also an increased overall risk to develop a neurodegenerative disorder such as Parkinson's Disease in astronauts is suspected (2, 8). Animal experiments using space-like radiation revealed impairments in memory, deficits in processing speed, attention and cognitive flexibility, as well as elevated anxiety levels and depressive behavior in mice (8, 9). The cellular mechanisms involved in these cognitive effects are currently under investigation. Animal studies demonstrated that increased cell death (10), decreased proliferation (11), increased DNA damage (12), cell cycle changes (13) and neuroinflammation including activation of microglia and astrocytes (14–16) might be involved in the response to space-relevant radiation qualities. Since cognitive detriments and increased risk of developing neurogenerative disease are crucial factors affecting astronaut health and mission success, further investigations of the underlying cellular and molecular processes are necessary. Astrocytes are the most abundant glial cells in the CNS and despite their supportive function in the physiological processes of the brain, they react under pathophysiological conditions with cellular, molecular and functional changes. This suggests that astrocytes play a crucial role in the brain's response to radiation.

The main impact of ionizing radiation on mammalian cells is damage to deoxyribonucleic acid (DNA), inducing breakage of both DNA strands (DNA double strand break, DSB), which could subsequently lead to cell death (17). Compared to low-LET radiation, high-LET radiation leads to dense ionizations along the particle tracks and induces more complex DNA damage which is difficult to repair (18). Cellular damage induced by ionizing radiation subsequently initiates an active cellular response, the DNA damage response, comprising DNA damage repair, altered gene expression, cell cycle arrest or programmed cell death (19–21). In general, while basic principles and mechanisms of the radiation response in mammalian systems are understood nowadays, tissue- or cell-type specific effects of ionizing radiation are still under investigation. For astrocytes, knowledge of their ability to repair DNA damage induced by ionizing radiation, especially heavy ion-induced DNA damage is scarce. In a seminal study comparing murine embryonic stem cell-derived neural stem cells and corresponding terminally differentiated astrocytes, astrocytes were radioresistant and expressed non-homologous end-joining genes enabling repair of ionizing radiation-induced DNA DSB (22). The repair kinetics of these DNA damages can indicate whether a cell-type is DNA repair-proficient or -deficient. Intracellular pathways are known to be activated in response to ionizing radiation, such as the nuclear factor κB (NF-κB) pathway. NF-κB is known to transcriptionally regulate a multitude of cellular responses, like immune response, inflammation *via* cytokine release, proliferation, cell cycle progression, and apoptosis. In other cell types, it was shown that the NF-κB subunit p65 translocates into the nucleus upon pathway activation in response to ionizing radiation exposure (20), including heavy ion exposure (21–23).

During pathophysiologic processes, e.g., CNS injuries, inflammation or exposure to toxic substances, astrocytes shift their phenotypic state from a normal naive state to a reactive state, also known as astrocyte reactivity. Depending on the severity of the nervous tissue insult, astrocytes become reactive, which spreads throughout the affected area as so-called reactive astrogliosis and ultimately leads to the formation of the glial scar (23, 24). This phenotypic change upon reactivity induction is accompanied by several traits, including hyperproliferation, increased cellular maintenance, morphological alterations, increased migration rates, cytokine release, and gene expression changes. Characteristic for astrocyte reactivity is the overexpression of the intermediate filament glial fibrillary acidic protein (GFAP) (23). Furthermore, astrogliosis is a heterogeneous process (23) with a continuous spectrum of severities (25). Depending on pleiotropic factors, astrocytes may maintain damage-induced inflammatory reactions and tissue damage or promote repair of tissue after becoming reactive (23). This process can also be triggered by neuroinflammation and plays a role in neurodegenerative mechanisms. Ionizing radiation is also known to induce neuroinflammation by microglia activation or astrocyte reactivity or by induction of radiation-induced senescence further promoting chronic inflammation (26, 27). In recent studies, stress response mechanisms in astrocytes include reactivity induction and cellular senescence (25), raising the question whether astrocytes respond with a transition into a reactive state or with so-called astrosenescence after exposure to ionizing radiation. Astrosenescence is characterized, for example, by a growth arrest, a senescence-associated secretory profile (SASP) involving increased secretion of cytokines such as interleukins (IL), as well as senescence-associated β-galactosidase activity, whereas astrocyte reactivity is accompanied by increased cytokine secretion in response to CNS insults (25).

Ionizing radiation is not the only risk factor astronauts face during space missions. Microgravity affects the human body by head ward fluid shift and mechanical unloading, resulting in changes in visual acuity (8), bone (28, 29) and muscle loss (30), reductions in plasma volume, cardiovascular deconditioning and neurovestibular alterations (31–36). In 30-day 6°-head-down-tilt bedrest study, changes in white and gray matter volume and white matter tracts of the brain of healthy volunteers were shown by magnetic resonance imaging (MRI) (37). Also, in animal models, microstructural alterations were found in multiple brain regions (37). On tissue level, in a biosatellite experiment with C57BL/6N mice, myelin degeneration of the sciatic nerve (38) and transcriptome changes were observed (39), and alterations in the choroid plexus were induced by hindlimb unloading or spaceflight in rats (40). Microgravity-induced effects are also observed on a cellular level (41, 42), for example, changes of organelles and the cytoskeleton (43–45), of migration (46–48), cell cycle regulation (49), cell proliferation (50), apoptosis (51), DNA repair (52), differentiation (8, 53, 54) and T cell regulation (41, 55), and gene expression, proteome and epigenetics alterations (49, 56, 57). Interestingly, increased mechanical loading in consequence to mild hypergravity exposure (2*g*) yielded an attenuation of astrocyte reactivity (58). Thus, astrocytes are sensitive to changes in gravity levels, but a clear understanding of the effects of multiple space-relevant conditions including microgravity and ionizing radiation on astrocytes is still missing. As DNA repair, cell cycle arrests, apoptosis and changes in proliferation and gene expression are hallmarks of the DNA damage response, the interaction of space radiation and microgravity effects on the cellular level needs to be understood also in astrocytes.

This study aims to characterize the response of primary murine astrocytes toward exposure to low- and high-LET radiation to further understand their role and function in the brain after radiation exposure. Primary murine astrocytes isolated from the cortex of mouse embryos are powerful tools to understand molecular pathways induced by radiation exposure and whether they secrete, e.g., cytokines (59). To determine if the DNA damage repair kinetics in astrocytes are comparable to other cell types, formation of phosphorylated H2AX (γH2AX) and p53 binding protein 1 (53BP1) foci was investigated *via* immunostaining after irradiation of primary murine astrocytes with different types of radiation. As arrest of cell cycle progression in response to ionizing radiation allows sufficient repair time of DNA damage, the cell cycle was analyzed and gene expression of regulators involved in different cell cycle check points (*Cdkn1a, Cdkn2a*) was studied *via* reverse transcription quantitative real-time Polymerase Chain Reaction (RT-qPCR). As the NF-κB pathway constitutes a major signaling pathway involved in inflammatory responses to ionizing radiation, its activation was investigated by immunostaining of the NF-κB subunit p65 and quantification of its nuclear localization. Astrocyte reactivity in response to ionizing radiation exposure was assessed by immunofluorescence staining of GFAP and the cell proliferation marker Ki-67. Since changes in gene expression are known to be part of astrocytes' reactivity, expression of *Gfap, Il1ß, Il6, Tgfß1*, and tumor necrosis factor (*Tnf*) was investigated by RT-qPCR. The transcriptomics profile of X-irradiated astrocytes was determined by mRNA sequencing. In order to differentiate reactivity from astrosenescence, cytokine secretion was quantified using enzyme-linked immunosorbent assays (ELISA). Furthermore, the effect of simulated microgravity on the repair of X-ray-induced DNA double strand breaks was analyzed using the principle of 2D fast clinorotation (60, 61) to gain a basic understanding of astrocytes' DNA damage response under space-like conditions.

## 2. Materials and methods

An overview of the experiments performed in this work indicating radiation qualities, doses and time points that were investigated for the different biological endpoints in primary murine astrocytes is given in Table 1.

**Table 1:** Overview of the experiments: radiation quality, doses, time points, biological endpoints.

**Biological endpoint conditions**		**Radiation qualities**			**Combined effects**
		**X-rays (200 kV, LET 0.3–3 keV/**μ**m)**	^12^**C (7 MeV/n, LET 220 keV/**μ**m)**	^56^**Fe (996.5 MeV/n, LET 151 keV/**μ**m)**	**Microgravity conditions**
DNA damage and repair	Doses (Gy)	0, 0.1, 1.0	Not determined	0, 0.1, 0.5, 1.0, 2.0	≤ 0.036*g*, 2 Gy
	Time points (h)	0, 0.5, 1.0, 4.0, 8.0, 24.0		1.0, 4.0, 8.0, 24.0	1.0, 4.0, 6.0, 24.0
Proliferation	Doses (Gy)	0, 2.0, 8.0	Not determined	Not determined	Not determined
	Time points (h)	0.5, 1.0, 4.0, 8.0, 24.0			
Cell cycle progression	Doses (Gy)	0, 8.0	Not determined	Not determined	Not determined
	Time points (h)	1.0, 2.0, 4.0, 6.0, 16.0, 24.0			
GFAP (astrocyte reactivity)	Doses (Gy)	0, 2.0, 8.0	Not determined	0, 0.1, 0.5, 1.0, 2.0	Not determined
	Time points (h)	0.5, 1.0, 4.0, 8.0, 24.0		1.0, 4.0, 8.0, 24.0	
NF-κB activation	Doses (Gy)	0, 2.0, 8.0, TNF-α (20 ng/ml)	Not determined	0, 0.1, 0.5, 1.0, 2.0	Not determined
	Time points (h)	0.5, 1.0, 4.0, 8.0, 24.0		1.0, 4.0, 8.0, 24.0	
Cytokine secretion	Doses (Gy)	0, 8.0	0, 0.5, 1.0, 2.0	Not determined	Not determined
	Time points (h)	1.0, 2.0, 4.0, 6.0, 16.0, 24.0	1.0, 2.0, 4.0, 6.0, 8.0, 16.0, 24.0		
Global gene expression	Doses (Gy)	0, 0.1, 2.0	Not determined	Not determined	Not determined
	Time points (h)	6.0, 24.0			
Expression of selected target genes	Doses (Gy)	0, 1.0, 4.0, 8.0	0, 0.5, 1.0, 2.0	0, 0.5, 2.0, 4.0	Not determined
	Time points (h)	2.0, 6.0, 16.0	2.0, 6.0, 16.0	2.0, 6.0, 16.0	

### 2.1. Preparation and cultivation of primary murine astrocytes

Primary murine astrocytes were isolated from cortices of C57BL/6J wildtype mouse embryos at embryonic day 18.5 (E18.5) as described in reference (58). This animal experiment was approved by the “Landesamt für Natur, Umwelt und Verbraucherschutz Nordrhein-Westfalen (LANUV)” (Office for Nature, Environment and Consumer Protection of North Rhine-Westphalia) in Recklinghausen, Germany, on December 4, 2017, under the file reference 84-02.04.2017.A319. Briefly, after pregnant mice were euthanized, embryos were taken out and brains were further dissected. Using a stereomicroscope, brain cortices were isolated by detaching them from meninges and hippocampi. Cortices were then incubated in 0.05 % Trypsin/HBSS (PAN Biotech, Aidenbach, Germany) for 15 min at 37 °C and subsequently washed 3 times with warm Hanks' Balanced Salt Solution (HBSS)/4-(2-hydroxyethyl)-1-piperazineethanesulfonic acid (HEPES) (Sigma Aldrich, St. Louis, MO, USA). Cells were further dissociated using a normal and a fire polished glass Pasteur pipet (Th. Geyer, Renningen, Germany). Cells from the cortices of all mouse embryos from one pregnant mouse were pooled. Finally, the single cell suspension was seeded into 75 cm^2^ Nunc™ EasYFlask™ cell culture flasks (cells of two to three brains per flask; ThermoFisher Scientific Waltham, MA, USA) in Minimum Essential Medium (MEM, PAN Biotech) containing 0.6 % glucose (Sigma Aldrich), 0.22 % NaHCO_3_ (Merck, Darmstadt, Germany), 2 mmol/L L-glutamine, MEM non-essential & essential amino acids (PAN Biotech), penicillin (100 U/ml)/streptomycin (0.1 mg/ml) (PAN Biotech) and 10 % fetal bovine serum (FBS, PAN Biotech), and cultured at 37 °C, 5 % CO_2_ and saturated humidity. Three days before an experiment, cells were trypsinized and seeded into suitable culture vessels (25 cm^2^ flasks, slide flasks or cover slips in 24-well-plates) at a density of 2 × 10^4^ cells/cm^2^ if not specified otherwise (passage 1). All experiments were performed with primary astrocytes in passage 1.

### 2.2. Irradiation

#### 2.2.1. X-rays

X-rays experiments (LET 0.3–3.0 keV/μm) were performed using the X-ray source RS225 (Gulmay Medical, now: X-Strahl, Surrey, UK) at DLR, Cologne, Germany. The X-ray tube was set to a voltage of 200 kV and a current of 15 mA. Using an ionizing chamber type TM30013 connected to dosimeter UNIDOS^webline^ (PTW, Freiburg, Germany) dose and dose rate were determined. A copper (Cu) filter with a thickness of 0.5 mm was used to eliminate soft X-rays. The dose rate was set to 1.0 Gy/min by adjusting the distance to the X-ray source with an electrically driven exposure table. Samples were irradiated at room temperature (RT). Mock-irradiated controls (0 Gy) were treated in the same way without turning the X-ray source on. After irradiation, samples were transferred to an incubator (37 °C, 5 % CO_2_ and saturated humidity) and harvested at different time intervals according to experimental requirements.

#### 2.2.2. Heavy ions

Exposure to ^56^Fe ions (1,000 MeV/n) was executed at the ring accelerator SIS 18 (“Schwerionensynchrotron 18”) at the GSI Helmholtzzentrum für Schwerionenforschung GmbH (GSI) in Darmstadt, Germany. Cells were irradiated in culture flasks upright positioned on a conveyor belt. Upright flasks were filled with 50 mL serum free α-MEM-medium (resulting in ≈1 % serum) to prevent desiccation during the irradiation procedure which lasted ~ 30 min.

Exposure to ^12^C ions (8.6 MeV/n) was performed at GSI Universal Linear Accelerator (UNILAC). Petri dishes with cells and medium were stored in a reservoir filled with prewarmed serum free α-MEM-medium. The reservoirs were placed in a plexiglass box next to the beamline exit window. The box had a large opening for the heavy ion beam. One petri dish at a time was then remotely retrieved by a robot and placed in the beamline for medium-free irradiation due to beam range limitations (Table 2).

**Table 2:** Characteristics of heavy ion beams.

**Ion species**	**Energy [MeV/n]**		**LET in water [keV/μm]**	**Range in water [μm]**	**Accelerator**
	**Beam**	**On target**			
Carbon (^12^C)	8.6	7.0	220	235	GSI UNILAC
Iron (^56^Fe)	1,000.0	996.5	151	266,700	GSI SIS

All samples were irradiated at room temperature. Mock-irradiated controls (0 Gy) were treated in the same way except for the turning on of the heavy ion beam. Dosimetry was performed by staff at accelerator facilities, and dose rates were adjusted to ≈1 Gy/min. The characteristics of the beams are listed in Table 2.

Fluence (F) was converted to dose by the Equation (1):


(1) $$\begin{array}{l} Dose\;\left[Gy\right]=1.6\times 10^{-9}\times LET\left[\frac{keV}{\mu m}\right]\times F\left[\frac{P}{cm^{2}}\right] \end{array}$$

To calculate average hits per cell nucleus, area of astrocyte nuclei was determined in formaldehyde-fixed DAPI-stained cells (Section 2.4). Photographs of stained nuclei were on Zeiss AxioObserver epifluorescence microscope (Carl Zeiss AG, Oberkochen, Germany) using Zen 3.0 blue software for imaging and analysis (Carl Zeiss AG). Average nucleus area (A) of astrocytes was 190.8 ± 67.2 μm^2^. The expected fluence (F_e_) per cell nucleus was calculated according to Equation (2):


(2) $$\begin{array}{l} F_{e}\left[\frac{P}{cell\;nucleus}\right]=10^{-8}\times A\left[{\mu m}^{2}\right]\times F\left[\frac{P}{cm^{2}}\right] \end{array}$$

Poisson distribution of heavy ion hits in cell nuclei was calculated according to Equation (3), and fractions of non-hit and hit cell nuclei were determined (Table 3).


(3) $$\begin{array}{l} f_{x}\left(x\right)=\frac{F_{e^{x}}}{x!}e^{{-F}_{e}},X=0,1,2,3,\ldots . \end{array}$$

**Table 3:** Hit calculation for carbon and iron ions exposure of primary murine astrocytes.

**Ion species**	**Fluence (P/cm^2^)**	**Dose (Gy)**	**Unhit fraction**	**Hit fraction**	**Average hits**
			**of the irradiated cell population**		**per cell nucleus**
Carbon (^12^C) 7.0 MeV/n	2.84E+05	0.1	0.58	0.42	0.5
	1.42E+06	0.5	0.07	0.93	2.7
	2.84E+06	1.0	0.00	1.00	5.4
	5.67E+06	2.0	0.00	1.00	10.8
	1.13E+07	4.0	0.00	1.00	21.7
Iron (^56^Fe) 996.5 MeV/n	4.15E+05	0.1	0.55	0.45	0.59
	2.07E+06	0.5	0.05	0.95	2.94
	4.15E+06	1.0	0.00	1.00	5.89
	8.30E+06	2.0	0.00	1.00	11.78
	1.66E+07	4.0	0.00	1.00	31.65

### 2.3. Simulated microgravity

Exposure to simulated microgravity was performed using a custom-build 2D fast rotating clinostat, specifically constructed for the adaptation of slide flasks (growth area 9.0 cm^2^, ThermoFisher Scientific, MA, USA). Simulation of microgravity in such ground-based facilities is based on randomization of the Earth's gravity vector in cells in culture. Exposure of the cells to simulated microgravity is performed in the slide flask mounted into the clinostat alongside the rotation axis. At the constant rotation speed of 60 rpm perpendicular to the direction of the gravity vector, all cells lying within three millimeters of the rotation axis center will perceive a calculated acceleration of ≤ 0.006*g*. The further away a sample is from the rotational axis, the higher residual *g*-forces it will be subjected to. The highest residual acceleration that could be perceived by the cells on the outmost regions of the slide flasks was calculated to ~0.036*g* (62). Simulated microgravity exposure is highly susceptible to disturbances by environmental stimuli, such as vibrations and shear forces. The clinostat was optimized to avoid vibrations and to be employed inside a cell culture incubator for optimal environmental conditions of 37 °C, >90 % relative humidity and 5 % CO_2_ with minimal vibrations during the exposure to simulated microgravity [validation see: Brungs et al. (45)]. The rotation speed was calculated for the respective vessels to apply optimal and highest-quality levels of microgravity. The slide flasks were filled completely with degassed cell culture medium and any remaining bubbles were removed before closing the flasks, to avoid any shear forces. The astrocytes in slides flasks were exposed to X-rays as described in Section 2.2.1 and directly mounted into the 2D clinostat within the incubator. Static 1*g* controls were exposed to 1*g* on top of the clinostat within the incubator. The cells were incubated at 37 °C and 5 % CO_2_ for up to 24 h after irradiation.

### 2.4. Immunofluorescence staining and fluorescence microscopy

For immunofluorescence staining, 1 × 10^4^ astrocytes were seeded on sterilized glass coverslips (Ø 10 mm, Carl Roth GmbH & Co.KG, Karlsruhe, Germany) or slide flasks (ThermoFisher Scientific) and grown for 3 days. Astrocytes were exposed to radiation as described in Section 2.2. For some biological endpoints (proliferation and NF-κB activation), recombinant murine TNF-α (20 ng/ml; Peprotech, Hamburg, Germany) was used as positive control and added to a separate batch of cells at the time of irradiation. Astrocytes were then cultivated in MEM-FBS until fixation. At respective time points, cells were fixed with 3.5 % formaldehyde (FA, Sigma Aldrich, USA) in phosphate-buffered saline (PBS) at 37 °C and 5 % CO_2_ for 30 min. Afterwards, FA was replaced by PBS and cells were stored at 4 °C until immunofluorescence staining was performed.

Cells were then permeabilized with 0.3 % Triton-X/PBS supplemented with 1 % DMSO and 5 % normal goat serum (NGS) for 1 h at RT. Primary antibodies were diluted in 0.3 % Triton/PBS + 1 % DMSO, slides were covered with antibody solution and incubated overnight at 4 °C in a wet chamber. After washing three times with PBS, cells were stained with secondary antibodies and 0.5 ng/ml 4′,6-diamidino-2-phenylindole (DAPI) at RT for 45 min. Finally, coverslips were washed and mounted onto glass slides (VWR, Darmstadt, Germany) using Fluoromount mounting medium (Agilent Dako, Santa Clara, CA, USA). The following primary antibodies were used: anti-GFAP antibody (1:500, Abcam, Cambridge, UK, #ab4674), Ki-67 (1:100, Abcam, #ab16667), anti-NF-κB p65 (1:250, Abcam, #ab32536), anti-H2A.X Phospho (Ser139) (1:1,000, Biolegend, Koblenz, Germany, # 613401) and anti-53BP1 antibody (1:1,000, Abcam, # ab21083). These secondary antibodies were used: goat anti-chicken Alexa Fluor 488 (1:1,000, Abcam, #ab150173), goat anti-rabbit Atto 550 (1:1,000, Merck KGaA, Darmstadt, Germany, #43328), goat anti-rabbit Atto 488 (1:1,000, Sigma Aldrich, #43328), goat anti-mouse Atto 488 (1:1,000, Sigma Aldrich) and goat anti-rabbit Atto 550 (1:1,000, Sigma Aldrich, #43328).

Immunostained cells were assessed microscopically with the Axio Observer.Z1 epifluorescence microscope (Carl Zeiss AG, Jena, Germany) using the Zen 3.0 blue software (Carl Zeiss AG). Exposure times were determined based on immunostainings with secondary antibody only for the highest applied dose or positive control and kept constant within the experimental set. Images were taken as two or four channel images at a magnification of 400 × , for which nine to twelve images of neighboring regions were taken as tile scans and stitched together in the Zen software. For each coverslip, three to five tile regions were imaged, these were then analyzed specifically for each respective experimental approach as described below. A minimum of 500 cells per sample were evaluated for each staining.

Analysis of DNA damage foci was performed with ImageJ. Total number of γH2AX and 53BP1 foci was determined by the local fluorescence maxima within a cell nucleus mask based on DAPI staining.

Proliferation of cells was analyzed in Zeiss Zen 3.0 software by first selecting all DAPI positive nuclei and then sub-selecting all Ki-67 positive nuclei. From the obtained data, the percentage of Ki-67 positive cells was calculated.

To quantify the reactive state of astrocytes, the GFAP immunostaining was analyzed using Zeiss Zen 3.0 software. Because GFAP is basally expressed in all astrocytes, a threshold for cells with upregulation of GFAP was set, as well as a minimal threshold for non-reactive cells with basal GFAP expression. Total number of cells for data normalization was determined by DAPI-stained nuclei counts. After definition of low and high GFAP expression thresholds, a size filter was applied to exclude regions below a minimum area of 80 μm^2^ as these might represent residual microglial cells. For further analyses, GFAP area in μm^2^ and fluorescence intensity [Grey] were chosen. Further calculations were done on Excel 2019 (Microsoft) by first normalizing the data to the region area and weighting them according to region area by following equation:


(4) $$\begin{array}{l} weighted\;intensity=\sum \left(intensity\times \;\frac{area}{\sum area}\right) \end{array}$$

Activation of NF-κB pathway was quantified by determining translocation of subunit p65 into the cell nucleus. In ImageJ, a cell nucleus mask was selected based on the DAPI staining. The intensity of the p65 fluorescence signal per pixel was measured in the cell nucleus mask. The raw integrated density was then calculated as the sum of the pixel intensities within the nucleus area. In Excel, the raw integrated density for each treatment was normalized to the raw integrated density of the untreated control at the earliest time point as is given as p65 fluorescence intensity in Grey.

### 2.5. Cell cycle analysis by flow cytometry

To determine the number of cells in the different cell cycle phases after irradiation, 1 × 10^4^ astrocytes per cm^2^ were seeded in Ø 6 cm cell culture dishes (LABSolute, Th. Geyer GmbH) (X-rays), 25 cm^2^ CytoOne cell culture flasks (STARLAB International GmbH, Hamburg, Germany) (GSI SIS) or Ø 3.5 cm NUNC™ EasY Dish cell culture dishes (ThermoFisher Scientific) (GSI UNILAC). Three days after seeding, cells were irradiated with different doses of X-rays (see Section 2.2.1) or heavy ions (Section 2.2.2), respectively. At chosen time points cells were washed and trypsinized with 0.05 % Trypsin/EDTA (PAN Biotech). The single cell suspension was fixed with 37 % FA for 30 min at 4 °C. After washing with PBS, 1 × 10^5^ cells per well were transferred into a 96-well MicroWell plate (Th. Geyer GmbH). After washing twice with PBS, cells were stained with DAPI (0.5 μg/ml) in 0.1 % Triton X-100 in PBS overnight at 4 °C. On the following day, cells were washed once with PBS and the DAPI fluorescence signal was measured in technical duplicates by flow cytometry (CytoFLEX S with the software CytExpert 2.5, Beckman Coulter, Indianapolis, USA) for a minimum of 10,000 cells per sample well and further analyzed with FlowJo™ (Becton, Dickson and Company, Franklin Lakes, USA). The gating strategy encompassed a side vs. forward scatter dot plot to exclude debris, an area vs. width dot plot of the DAPI channel (PB450) to exclude doublets. From the PB450 histogram displaying single cells, the percentage of cells in G1, S-phase and G2 phase of the cell cycle was computed.

### 2.6. Gene expression analysis

#### 2.6.1. RNA sequencing

The global transcription profile after exposure to X-rays was analyzed by mRNA sequencing. Primary murine astrocytes were seeded in cell culture dishes (6 cm) from confluent 75 cm^2^ tissue culture flasks (Nunc™) at a density of 2 × 10^4^ cells/cm^2^. Three days after seeding, cells were irradiated with 0, 0.1 and 2 Gy of X-rays as described in Section 2.2.1. For harvest 6 h or 24 h after irradiation, medium was completely removed and cells were lysed using RLT buffer (Qiagen) with β-mercaptoethanol (1:100, Sigma Aldrich). The homogenized lysate was stored at −80 °C until RNA isolation with RNeasy Mini Kit on the same day for all samples. RNA concentration and integrity were determined by means of the RNA 6000 Nano Assay in the Bioanalyzer (Agilent Technologies, Böblingen, Germany). RNA Integrity Number (RIN) of all samples was above 9.0. At least 3 μg total RNA per sample (4 biological repeats per condition) were sent on dry ice to GENEWIZ (Leipzig, Germany) for mRNA sequencing in the same run after Poly(A) selection using the Illumina NovaSeq6000 platform (configuration: 2 × 150 bp, 350 M read pairs) and bioinformatics analysis including trimming, mapping, and differential gene expression following principles described in Koch et al. (63). Significantly differentially expressed genes were clustered by their gene ontology (GO) and the enrichment of GO terms was tested using Fisher exact test (GeneSCF v1.1-p2).

#### 2.6.2. Reverse Transcriptase quantitative real-time Polymerase Chain Reaction (RT-qPCR)

Reverse Transcriptase quantitative real-time Polymerase Chain Reaction (RT-qPCR) was used to determine expression of selected target genes (*Cdkn1a, Cdkn2a, Gfap, Il1ß, Il6, Tfgß1*, and *Tnf*) in comparison to housekeeping gene hypoxanthine-guanine-phosphoribosyl-transferase 1 (*Hprt-1*) (Table 4). Astrocytes were seeded in Ø 6 cm cell culture dishes (LABsolute, Th. Geyer GmbH) at a density of 5 × 10^4^ cells/cm^2^ for X-irradiation (see Section 2.2.1). For heavy ion irradiation (see Section 2.2.2) at GSI SIS, cells were seeded on 25 cm^2^ CytoOne^®^ cell culture flasks (STARLAB International GmbH), and for irradiation at GSI UNILAC, cells were seeded in Ø 3.5 cm Nunc™ EasY Dish cell culture dishes (ThermoFisher Scientific). Cells were irradiated 3 days after seeding and extraction of ribonucleic acid (RNA) was performed by using RNeasy Mini Kit (Qiagen, Hilden, Germany). RNA concentration and integrity were measured with the RNA 6000 Nano Assay (Agilent Technologies, Santa Clara, CA, USA) according to the manufacturer's protocol. Complementary deoxyribonucleic acid (cDNA) was synthesized from 1 μg RNA per sample in a volume of 80 μl using the iScript cDNA Synthesis Kit (Bio-Rad, Feldkirchen, Germany) which contains a mixture of oligo (dT) and random primers. Finally, qPCR analysis was performed in technical duplicates using QuantiFast SYBR Green PCR Kit (Qiagen) and the CFX96 Deep Well Optics System (Bio-Rad). For each target gene, the concentrations of forward and reverse primer were optimized using final primer concentrations of 0.5–10 μmol/l. The optimized primer concentrations are listed in Table 4. The temperature protocol of the qPCR reaction was: 5 min initial denaturation at 95 °C followed by 40 cycles of denaturation for 10 s at 95 °C and annealing and extension at 60 °C for 30 s, and melting curve protocol. Relative quantity, relative expression and fold change of gene expression were determined by the efficiency-corrected ΔΔCT method.

**Table 4:** Primers for RT-qPCR.

**Gene name abbreviation**	**Sequence (5** ^**′**^ **-3** ^**′**^ **)**		**Final concentration in reaction mix [μmol/l]**	**Volume per reaction mix [μl]**	**Gene identifier, gene ID**
**Housekeeping gene**					
*Hprt*	Fwd	AGGGATTTGAATCACGTTTG	1.0	0.25	15452
	Rev	TTTACTGGCAACATCAACAG	1.0	0.25	
**Target genes**					
*Cdkn1a*	Fwd	ACCTGATGATACCCAACTAC	1.0	0.25	12575
	Rev	CTGTGGCACCTTTTATTCTG	2.0	0.50	
*Cdkn2a*	Fwd	ACTCCAAGAGAGGGTTTTC	4.0	1.00	12578
	Rev	ATCATCATCACCTGGTCC	1.0	0.25	
*Gfap*	Fwd	GGAAGATCTATGAGGAGGAAG	2.0	0.50	14580
	Rev	CTGCAAACTTAGACCGATAC	4.0	1.00	
*Il1β*	Fwd	GGATGATGATGATAACCTGC	4.0	1.00	16176
	Rev	CATGGAGAATATCACTTGTTGG	4.0	1.00	
*Il6*	Fwd	AAGAAATGATGGATGCTACC	2.0	0.50	16193
	Rev	GAGTTTCTGTATCTCTCTGAAG	1.0	0.25	
*Tgfβ1*	Fwd	GGATACCAACTATTGCTTCAG	2.0	0.50	21803
	Rev	TGTCCAGGCTCCAAATATAG	1.0	0.25	
*Tnf*	Fwd	CTATGTCTCAGCCTCTTCTC	1.0	0.25	21926
	Rev	CATTTGGGAACTTCTCATCC	1.0	0.25	

### 2.7. Cytokine detection

Free IL-6 concentration in cell supernatants was determined using the IL-6 Mouse Uncoated ELISA Kit (#88-7064-22, Invitrogen, ThermoFisher Scientific) according to the manufacturer's instructions. Directly after irradiation of astrocytes, cell culture medium was renewed. Cell culture media supernatants (1,000 μL) were taken at chosen time points after exposure when cells were harvested for other endpoints (Sections 2.4, 2.5, and 2.6) and stored at −80 °C until IL-6 was quantified using ELISA. The quantification was performed with technical duplicates. The color reaction was detected with Multiskan™ FC Microplate Photometer (ThermoFisher Scientific) and the Skanit™ Software 3.1 (ThermoFisher Scientific). Analysis of the data was done in Excel 2019 (Microsoft) by standard reference curve.

### 2.8. Statistics

The number of independent experiments is indicated in the figure legends, and the number of technical repetitions is specified in the respective method section. For X-rays experiments, at least three independent experiments were performed. Each heavy ion beamtime could only be performed once, but for some endpoints, independent irradiations of astrocytes from different animals were implicated. These data from independent experiments are represented as mean and respective standard deviation. Statistical tests were done with GraphPad Prism 6.0 (GraphPad, San Diego, CA, USA), including *t*-test and two-way ANOVA.

## 3. Results

Experiments performed in this work showed that astrocytes repair ionizing radiation-induced DNA double strand breaks at a pace comparable to other cell types. Their radiation response is quite subdued without prominent changes in cell cycle progression and gene expression. The reaction profile trends toward senescence, and a pronounced induction of reactivity was not observed.

### 3.1. DNA damage and repair

**Figure 1:**
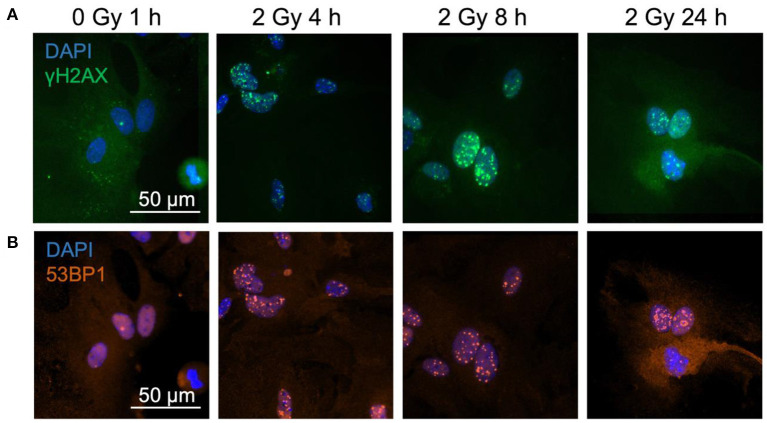
γH2AX and 53BP1 foci form in astrocytes' nuclei after exposure to iron ions. Astrocytes were mock-irradiated (0 Gy) or irradiated with 2 Gy of ^56^Fe ions and fixed at different time points for immunofluorescence staining of the DNA double strand marker γH2AX and the DNA repair protein 53BP1. 53BP1 as a marker protein expressed mainly during non-homologous end-joining (NHEJ) was assessed as indicator of DNA repair pathway choice. Cell nuclei were stained with DAPI (blue) for all images. **(A)** Exemplary images of γH2AX (green) immunostaining of astrocytes. Scale bar: 50 μm. **(B)** Exemplary images of 53BP1 (red) immunostaining. Bar: 50 μm.

To analyze radiation-induced DNA damage and subsequent repair, primary murine astrocytes were exposed to different doses of X-rays and iron (Fe) ions. As DNA double strand breaks (DSBs) are considered to be strongly detrimental compared to other DNA damages, cells were stained against the phosphorylated Histone 2AX (Figure 1A) which is present in chromatin surrounding DNA DSBs, and additionally for the DNA-repair associated protein 53BP1 (Figure 1B). To determine the kinetics of radiation-induced DNA DSBs and their repair, fixation time points up to 24 h after irradiation were included. For determination of a dose- and radiation quality-dependence of DSBs induction and repair, different doses as well as different radiation qualities were considered.

**Figure 2:**
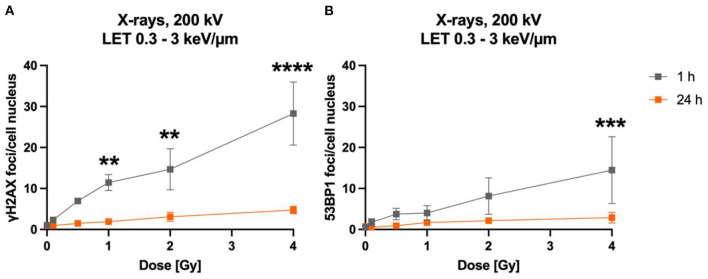
The number of γH2AX and 53BP1 foci formed in X-irradiated astrocytes increases dose-dependently. Astrocytes were mock-irradiated (0 Gy) or irradiated with X-ray doses (200 kV, 15 mA) up to 4 Gy and fixed 1 and 24 h after irradiation for immunofluorescence staining of the DNA double strand marker γH2AX **(A)** and the DNA repair protein 53BP1 **(B)**. The number of foci (γH2AX, 53BP1) per cell nucleus was quantified to generate dose effect curves of the initial foci number (1 h) and the number of foci after a repair time of 24 h. The sample size was *n* = 3 for both immunofluorescence stainings. The samples were compared *via* 2way ANOVA (Tukey's multiple comparisons test), based on a sample size *n* = 3. Data are shown as mean ± SD. In case that the error bars are smaller than the symbol, they are not visible. Significant differences in comparison to the respective 0 Gy control are indicated by asterisks (**p < 0.01, ***p < 0.001, ****p < 0.0001).

In a first approach, the dose-dependency of γH2AX and 53BP1 foci formation 1 h after exposure to X-rays and the number of foci after a repair time of 24 h were determined (Figure 2). The dose-effect curve of γH2AX foci in X-irradiated astrocytes indicated a dose-dependent induction of DNA DSBs at 1 h; low levels of γH2AX foci remained after 24 h repair time (Figure 2A). Similarly, 53BP1 foci accumulated dose-dependently in astrocytes 1 h after X-irradiation, and levels of 53BP1 foci per cell nucleus were reduced after 24 h (Figure 2B). The total number of 53BP1 foci 1 h after irradiation was lower than the number of γH2AX foci at this time point.

**Figure 3:**
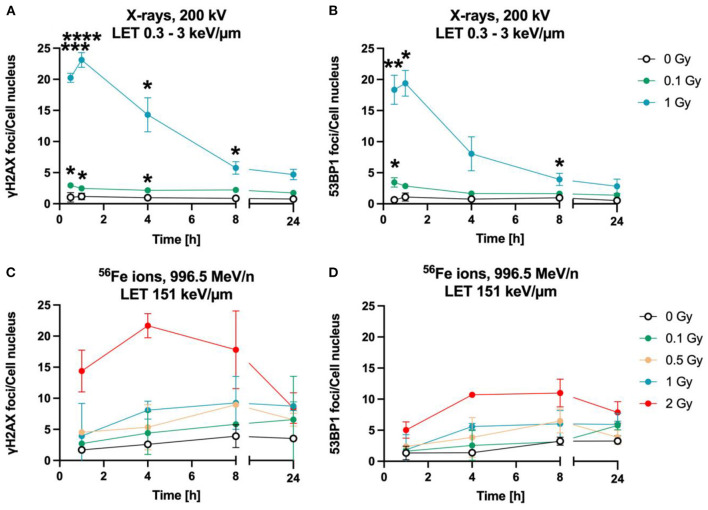
Astrocytes encounter DNA double strand breaks after exposure to X-rays and iron ions and are fully repair-proficient *via* non-homologous end-joining. Astrocytes were mock-irradiated (0 Gy) or irradiated with X-rays (200 kV, 15 mA) with doses up to 1 Gy (upper panel) or Fe ions (LET 151 keV/μm, 996.5 MeV/n) with doses up to 2 Gy (lower panel). They were fixed at different time points up to 24 h after irradiation for immunofluorescence staining of the DNA double strand marker γH2AX (left) and the DNA repair protein 53BP1 (right). **(A)** Number of γH2AX foci per cell nucleus after X-irradiation. **(B)** NHEJ activity was quantified over 24 h by the number of 53BP1 foci in astrocytes' nuclei exposed to different doses of X-rays. **(A, B)** The samples were compared *via* 2way ANOVA (Tukey's multiple comparisons test), based on a sample size *n* = 3. Significant differences in comparison to 0 Gy control are indicated by asterisks (*p < 0.05, **p < 0.01, ***p < 0.0001, ****p = 0.0001). Cells showed a dose- and time dependent accumulation of 53BP1 foci. **(C)** Number of γH2AX foci per astrocyte nucleus irradiated with different doses of ^56^Fe ions (*n* = 2). **(D)** Number of 53BP1 foci per cell after irradiation of astrocytes with different doses of ^56^Fe ions (*n* = 2). Data are shown as mean ± SD. In case that the error bars are smaller than the symbol, they are not visible **(B, D)**.

The time-dependency of DNA DSBs induction and decrease was investigated over a time period of up to 24 h for heavy ions and X-rays (Figure 3). Exposure to lower doses (0.1 Gy) and to 1 Gy of X-rays (LET 0.3–3 keV/μm) increased the number of γH2AX (Figure 3B) and 53BP1 (Figure 2B) foci per cell nucleus at 1 h after irradiation. After irradiation with 1 Gy X-rays, a maximum of ~23 γH2AX and ~20 53BP1 foci/nucleus was reached at this time point. Subsequently, the amount of γH2AX- and 53BP1 foci decreased over time until only minor fractions of γH2AX foci were present 24 h after irradiation, which were still higher compared to non-irradiated cells for the higher doses, but without reaching statistical significance.

The foci kinetics that were obtained for cells irradiated with ^56^Fe ions (LET 151 keV/μm) (Figures 3C, D) were delayed compared to X-rays. The number of γH2AX foci showed a trend to increase dose-dependently with a maximum 4 h (^56^Fe, ~22 foci/nucleus) after irradiation and to subsequently decrease to near the baseline (Fe ions) after 24 h. Comparing the early fast repair during the first 4–8 h after irradiation reveals a lower reduction in the foci number after iron ion exposure compared to X-rays exposure indicating slower repair kinetics after heavy ion irradiation. After Fe ion exposure, the number of foci at 24 h is still above the mock-irradiated control (Figure 3C). The number of 53BP1 foci showed a trend for a dose-dependent increase with a maximum after 4 to 8 h for cells irradiated with ^56^Fe ions (Figure 3D).

**Figure 4:**
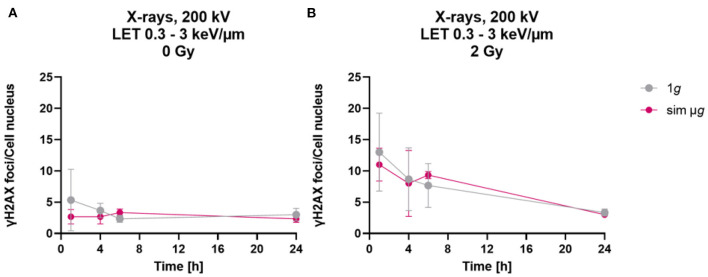
Simulated “space conditions” using simulated microgravity combined with X-irradiation did not influence repair of DNA double strand breaks. The number of γH2AX foci per cell of unirradiated **(A)** or X-irradiated (2 Gy) **(B)** astrocytes exposed after irradiation to either simulated microgravity (sim μ*g*) by fast 2D clinorotation at 60 rpm or at normal 1*g* gravity conditions. The samples were compared *via* two-way-ANOVA (*n* = 3, *p* < 0.05). Data are shown as represent mean ± SD.

When astrocytes were exposed to simulated microgravity following exposure to X-rays, a comparable number of γH2AX foci was observed 1 h after exposure to 2 Gy X-rays under static incubation and 2D clinorotation (Figure 4B). The absolute number was smaller than expected from the results shown in Figures 2A, 3A. This might have been caused by the different culture vessels that had to be used for the 2D clinostat experiments. In X-rays only experiments (Figures 2A, 3A), astrocytes were cultivated on cover slips to ensure optimal fluorescence microscopy. In the 2D clinostat, slide flasks with a polystyrene bottom had to be used as these can be closed tightly and filled with medium without air bubbles (Figure 4B). This might have affected the staining and foci counting process. No significant differences in γH2AX foci numbers were observed between the 1*g* control cells and cells exposed to simulated microgravity (sim μ*g*) for both, unirradiated (Figure 4A) and 2 Gy X-rays-exposed cells (Figure 4B). As no modulating effects of simulated microgravity on repair of DNA double strand breaks induced by exposure to X-rays were observed, no experiments attempting to combine exposure to heavy ions and simulated microgravity were made.

### 3.2. Cell cycle progression is slow and barely affected by exposure to X-rays

**Figure 5:**
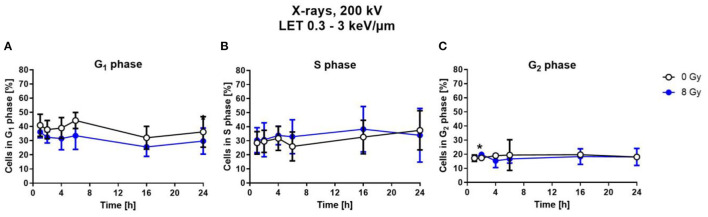
Exposure to X-rays did not induce cell cycle arrests. Astrocytes were exposed to 8 Gy X-rays or mock-irradiated (0 Gy) and trypsinized and fixed at different time points up to 24 h after irradiation. The percentage of cells in the different cell cycle phases—G_1_- **(A)**, S- **(B)**, G_2_-phase **(C)**— was determined by flow cytometry of DAPI stained cells. The distribution of cells in S- and G_2_-phase is comparable for both conditions. The samples were compared by unpaired *t*-test with a sample size of *n* = 6. Data are shown as mean ± SD. Significant differences in comparison to the 0 Gy control at the same time point are indicated by asterisks (*p < 0.05).

To determine cell cycle distribution, nuclear DNA was measured *via* DAPI fluorescence of single cells by flow cytometry. Based on this, the number of cells in the respective cell cycle phases was calculated. For this, astrocytes were exposed to 8 Gy of X-rays, and analyzed at different fixation time points between 1 h and 24 h after irradiation (Figure 5).

Compared to X-ray irradiated cells, in the unirradiated control, a slightly higher number of cells was in G_1_ phase (Figure 5A), without reaching statistical significance. The percentage of cells in the S-Phase fluctuated around 30 % over the time post-irradiation for both, X-rays-irradiated and unirradiated cells (Figure 5B). The number of cells in the G_2_ phase is nearly constant at about 10–15 % for all time points for both conditions (Figure 5C).

### 3.3. Astrocytes' proliferation is largely unaffected by exposure to X-rays

**Figure 6:**
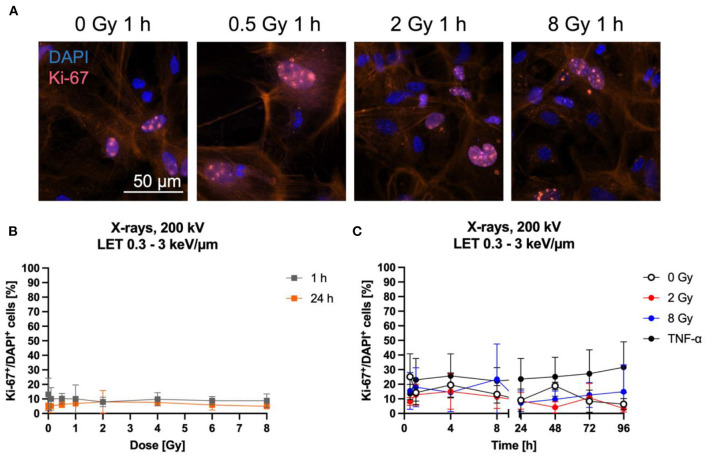
Proliferation of astrocytes remained mainly unaffected by radiation exposure. Astrocytes were exposed to X-rays (200 kV, 15 mA) with doses up to 8 Gy and fixed for Ki-67 immunostaining after different time points. Ki-67 is an indicator of proliferating cells. **(A)** Exemplary images of Ki-67 (pink) immunostaining showing an unirradiated control and cells 1 h after exposure to different doses of X-rays. The nucleus was stained with DAPI (blue) for all images, resulting in blue (Ki-67 negative) or blueish-pink cell nuclei with pink Ki-67 spots (Ki-67 positive, Ki-67^+^). The red fluorescence in the cytoplasm is due to phalloidin-647 staining of actin. Bar: 50 μm. **(B, C)** Quantitative evaluation of the Ki-67^+^DAPI^+^ cells after exposure of astrocytes to X-rays. **(B)** Dose effect curves of Ki-67^+^ cells 1 h and 24 h after exposure to X-rays (*n* = 4). **(C)** Kinetics of Ki-67^+^ cells up to 96 h after exposure to X-rays. TNF-α was added to unirradiated samples at the time of irradiation at a concentration of 20 ng/ml as it was previously described to stimulate proliferation of astrocytes. The percentage of Ki-67^+^ cells remained mainly unaffected by irradiation with different doses of X-rays over a time period of 1 h to 96 h. The samples were compared *via* one-way ANOVA, based on a sample size *n* = 4 (*p* < 0.05). Data are shown as mean ± SD.

Astrocyte proliferation is in general a measure of their reactivity (64–66). Furthermore, cell proliferation can be influenced by NF-κB pathway activation (67–69). Both can be induced by ionizing radiation (70–73) and are therefore of interest to further characterize the radiation response of astrocytes. Here, proliferation after exposure of astrocytes to X-rays was analyzed by immunostaining of the proliferation marker Ki-67 (Figure 6A) and quantification of Ki-67^+^ cells (Figures 6B, C). The number of Ki-67^+^ cells did not change 1 h and 24 h after X-irradiation with doses between 0.5 and 8 Gy—the number of Ki-67^+^ cells remained constant at around 10 % for all doses (Figure 6B). To follow Ki-67 expression over a longer time period after exposure to X-rays, astrocytes were exposed to 0, 2 and 8 Gy X-rays and Ki-67 was investigated over a time period of 96 h. The fraction of Ki-67^+^ cells varied between ~5 and ~30 % for all doses and time points (Figure 6C). No significant effects on Ki-67 expression were found for X-rays exposure up to 8 Gy. TNF-α was previously described to stimulate proliferation of primary astrocytes of several species (74–79) and was therefore used as positive control for proliferation stimulation. In primary astrocytes from 1- to 2-day old rats, the maximal stimulation of proliferation was observed following treatment with 10 ng/ml TNF-α (77), while in human astrocytes, 50 ng/ml human TNF-α increased the fraction of Ki-67^+^ cells significantly (79), and in simian astrocytes, the maximal stimulation of proliferation was observed for 7.6 ng/ml human TNF-α (74). Therefore, an intermediate concentration of 20 ng/ml TNF-α was chosen in this work. This treatment increased the number of Ki-67^+^ astrocytes, reaching 25–30 %, being more than 2-times higher than in unirradiated or irradiated astrocytes but not significant due to the large standard deviation (Figure 6C).

### 3.4. Expression of glial fibrillary acidic protein (GFAP)

**Figure 7:**
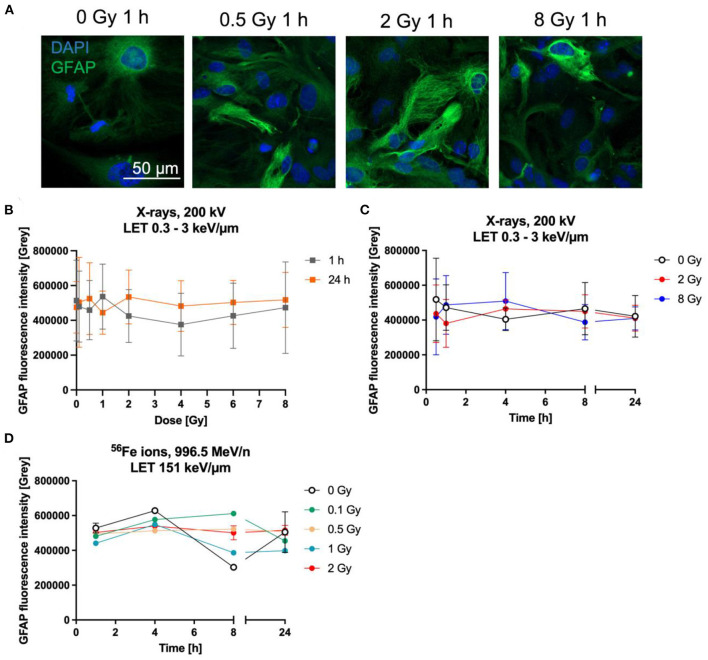
Astrocytes did not become reactive after radiation exposure. Astrocytes were exposed to X-rays (200 kV, 15 mA) with doses up to 8 Gy **(A–C)** or to iron ions **(D)** and fixed for GFAP immunostaining after different time points. **(A)** Exemplary microscopy images of GFAP (green) immunostaining showing an unirradiated control and cells irradiated with different doses of X-rays at 1 h after radiation exposure. The nucleus was stained with DAPI (blue) for all images. Bar: 50 μm. **(B)** Dose effect curves of astrocyte reactivity (GFAP) 1 h and 24 h after exposure to X-rays (*n* = 5). **(C)** The GFAP fluorescence intensity of astrocytes irradiated with different doses of X-rays over a time course up to 24 h reveals a basal expression of GFAP but no radiation-induced changes. The samples were compared *via* 2way ANOVA, based on a sample size *n* = 4 (*p* < 0.05). **(D)** Exposure to different doses of ^56^Fe ions (LET 151 keV/μm, 996.5 MeV/n) did not lead to a dose-dependent increase of GFAP expression, but astrocytes showed a basal expression (*n* = 2). In **(B–D)** data are shown as mean ± SD. In case that the error bars are smaller than the symbol, they are not visible **(D)**.

One commonly used marker to determine the reactivity of astrocytes is GFAP. The expression of GFAP was determined by the measurement of fluorescence intensity of GFAP-immunostained astrocytes (Figure 7A) up to 24 h after irradiation with X-rays or heavy ions. One h and 24 h after irradiation with up to 8 Gy X-rays, GFAP did not increase dose-dependently (Figure 7B). In order to detect possible transient GFAP increases, regulation over time after irradiation with 2 and 8 Gy of X-rays was determined, using fixation times between 0.5 h and 24 h (Figure 7C). GFAP expression after exposure to X-rays did not significantly change between unirradiated and irradiated cells. In all conditions, the basal intensity was around 500,000 and stayed in this range (Figures 7B, C).

In a second approach, the GFAP fluorescence after irradiation with Fe ions was followed for up to 24 h. Astrocytes irradiated with ^56^Fe (Figure 7D) ions showed a similar response compared to cells irradiated with X-rays, revealing that most cells express basal GFAP levels and only a few showing higher staining intensity, without clear differences between the doses.

### 3.5. NF-κB activation and cytokine secretion

**Figure 8:**
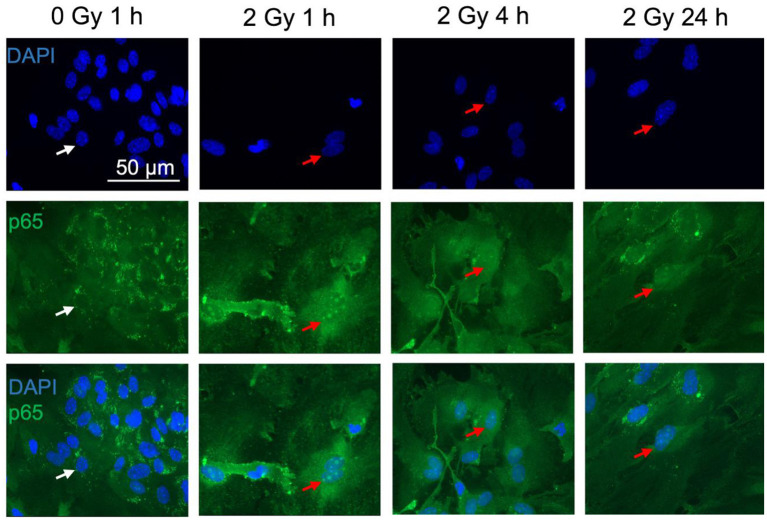
The NF-κB subunit p65 is detected in astrocytes. Astrocytes were exposed to 0 Gy (mock-irradiated control) or 2 Gy C ions (7 MeV/n, LET 220 keV/μm) and fixed 1, 4, and 24 h after treatment for immunofluorescence staining of the NF-κB subunit p65 (green). p65 is known to translocate from the cytoplasm into the nucleus upon NF-κB pathway activation. For visualization of the nuclear translocation, nuclear staining with DAPI (blue) is displayed in the **upper** panel, p65 immunofluorescence in the **middle** panel, and merged pictures of DAPI and p65 are shown in the **lower** panel. White arrows mark cells without p65 translocation in the nucleus. Red arrows indicate cells with p65 nuclear translocation. Scale bar: 50 μm.

**Figure 9:**
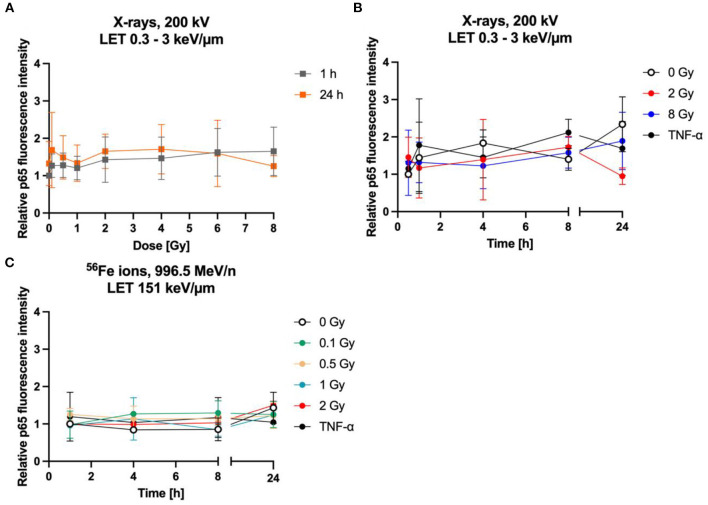
The NF-κB pathway is not activated by radiation exposure in astrocytes. Astrocytes were exposed to X-rays **(A, B)** or iron ions **(C)** and fixed at time points up to 24 h after irradiation for immunofluorescence staining of the NF-κB subunit p65. **(A)** Dose effect curves of activation of NF-κB pathway (p65) after exposure to X-rays (*n* = 6). **(B)** The relative fluorescence intensity of p65 in the nucleus area for different doses of X-irradiation over a time period up to 24 h, showing a basal activity of the NF-κB pathway but no further significant induction by radiation exposure or incubation with TNF-α. The samples were compared *via* Tukey's multiple comparison test, based on a sample size of *n* = 3. **(C)** Exposure to different doses of ^56^Fe ions (LET 151 keV/μm, 996.5 MeV/n) did not induce any further activation of NF-κB pathway (*n* = 2). The relative fluorescence intensity of p65 in the nucleus area was calculated by normalizing the raw integrated density for each treatment to the raw integrated density of the untreated control at the earliest time point that was investigated. Data are shown as mean ± SD.

In general, activation of the NF-κB pathway induces transcription of several genes such as the cytokine IL-6. It is well-known that IL-6 is predominantly produced by neurons and glial cells such as astrocytes in the CNS and plays an important role in the cell-cell-communication and astrocyte reactivity (80, 81). Regarding this, activation of the NF-κB pathway was studied by measurement of the fluorescence intensity of the NF-κB subunit p65 after immunostaining (Figures 8, 9) as well as of the IL-6 release into the cell culture supernatants of irradiated astrocytes using ELISA.

Upon NF-κB activation, the transcription factor dimer translocates from the cytoplasm into the cell nucleus. In case of ionizing radiation-induced NF-κB activation, the p65:p50 dimer as part of the proinflammatory pathway is involved amongst other NF-κB dimers. Therefore, localization of p65 in the cell nucleus was visualized and quantified after immunofluorescence staining. In mock-irradiated cells, diffuse and spotted p65 immunofluorescence was observed in the cytoplasm, while the spots where predominantly located in the perinuclear area (Figure 8, 0 Gy 1 h). A diffuse green fluorescence of lower intensity is also visible in some cell nuclei (Figure 8, 0 Gy 1 h, middle). After exposure to 2 Gy C ions, green spots appeared in some astrocytes' nuclei, and the cytoplasmic perinuclear spots seem to be reduced 1 h and 4 h after irradiation (Figure 8, 2 Gy 1 & 4 h), while partly reappearing at 24 h (Figure 8, 2 Gy 24 h). The nuclear spots were most prominent 1 h after exposure to C ions and seem to fade over the following hours.

Quantification of the nuclear green fluorescence intensity indicating nuclear p65 did not reveal a dose-dependent activation of the NF-κB pathway (Figure 9A). No significant changes of the fluorescence intensity of p65 in the area of the cell nucleus of astrocytes were observed up to 24 h after irradiation with X-rays (2 Gy, 8 Gy, Figure 9B) or heavy ions (Figure 9C). Also, treatment with TNF-α did not significantly increase nuclear localization of p65.

**Figure 10:**
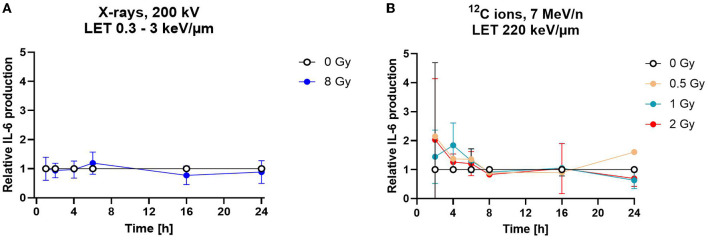
Astrocytes secrete the cytokine interleukin-6 independently of radiation exposure. Secretion of cytokine IL-6 was measured by ELISA. **(A)** The relative IL-6 concentration of in cell culture media supernatants is displayed for astrocytes irradiated with 8 Gy of X-rays over a time course of 1–24 h. The samples were compared *via* 2way-ANOVA (not significant, *n* = 5). **(B)** Astrocytes exposed to different doses of ^12^C ions (LET 220 keV/μm, 7 MeV/n) did not secrete more IL-6 than the mock-irradiated controls (0 Gy) for all conditions tested. The samples were compared by two-way-ANOVA (not significant, *n* = 4). Relative IL-6 concentrations were obtained by normalization of the absolute IL-6 concentration in the supernatants of the irradiated samples to the concentration in the supernatant of the mock-irradiated control (0 Gy) at each time point. Data are shown as mean ± SD for **(A, B)**. In case that the error bars are smaller than the symbol, they are not visible **(A, B)**.

To determine the release of IL-6 into cell culture supernatants, astrocytes were exposed to 8 Gy of X-rays or to doses of up to 2 Gy of C ions and supernatants were collected 1–24 h after irradiation (Figure 10). The average IL-6 production rate derived from the last time point of the mock-irradiated controls was 37 pg/h in the X-rays experiment and 97 pg/h in the C ion (7 MeV/n) experiment. After irradiation, the IL-6 secretion did not change significantly within the 24 h observation period (Figures 10A, B).

### 3.6. Radiation-induced gene expression

#### 3.6.1. Global gene expression

**Figure 11:**
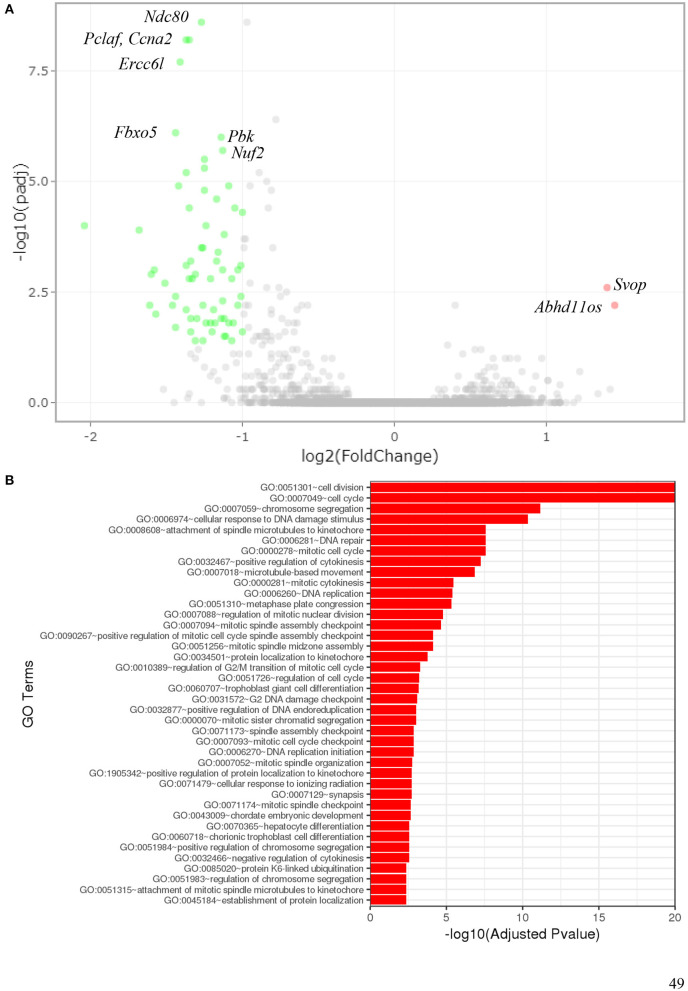
Gene expression of astrocytes 24 h after exposure to 2 Gy X-rays. Gene expression of primary murine astrocytes was analyzed using RNA sequencing (*n* = 4). **(A)** In this volcano plot, the log_10_ of the adjusted *P*-value [log_10_ (padj)] is plotted against the log_2_ Fold Change in expression of each gene. The two upregulated genes (pink dots) are annotated as well as some of the most significantly downregulated genes (green dots). **(B)** Gene ontology (GO) terms that were significantly enriched with an adjusted *P*-value < 0.05 in the differentially expressed gene sets are listed (up to 40 terms). The full list of enriched GO terms is available in Supplementary material.

To determine which pathways might be activated by ionizing radiation exposure of primary murine astrocytes, mRNA sequencing was performed with RNA isolated from astrocytes 6 and 24 h after exposure to 0.1 and 2 Gy X-rays. After exposure to 0.1 Gy X-rays, no genes were differentially regulated at both time points, 6 h and 24 h. Exposure to 2 Gy X-rays did not affect gene expression 6 h after irradiation; at 24 h, 68 genes were differentially expressed (Table 5; Figure 11A), of which two genes were upregulated (Table 6) and 66 genes were downregulated (Table 7). One of the upregulated genes, synaptic vesicle 2-related protein (*Svop*), is involved in synaptic vesicle transport, whereas the function of the second gene, the long non-coding RNA *Abhd11os*, is not yet categorized (Table 6). The downregulated genes are mainly involved in cell cycle control, proliferation, mitosis, cytokinesis and DNA repair and replication (Table 7). Testing for gene ontology [GO (82)] enrichment revealed 371 GO terms which are listed in Supplementary material. The 40 most significantly enriched GO terms are summarized in Figure 11B, and the 4 most enriched GO terms identify gene groups for: cell division, cell cycle, chromosome segregation and cellular response to DNA damage.

**Table 5:** Number of significantly differentially expressed genes (DEGs) in murine astrocytes after exposure to X-rays.

**Time after irradiation**	**Number of significant DEGs**			
	**0.1 Gy**		**2 Gy**	
	**Upregulated**	**Downregulated**	**Upregulated**	**Downregulated**
6 h	0	0	0	0
24 h	0	0	2	66

**Table 6:** Significantly upregulated genes in murine astrocytes 24 h after exposure to 2 Gy X-rays.

**Gene ID**	**Gene name**	**log_2_ fold change**	***P* adj**	**Function**
ENSMUSG00000042078	Svop	1.40	2.61E-03	Synaptic vesicle transport
ENSMUSG00000085042	*Abhd11os*	1.45	5.85E-03	Unknown

*P* adj, adjusted *P*-value.

**Table 7:** Significantly downregulated genes in murine astrocytes 24 h after exposure to 2 Gy X-rays.

**Gene ID**	**Gene name**	**log_2_ fold change**	***P* adj**	**Function**
ENSMUSG00000024056	*Ndc80*	−1.27	2.47E-09	Proliferation
ENSMUSG00000027715	*Ccna2*	−1.35	6.41E-09	Proliferation
ENSMUSG00000040204	*Pclaf*	−1.37	6.41E-09	Proliferation
ENSMUSG00000051220	*Ercc6l*	−1.41	1.96E-08	Mitosis
ENSMUSG00000019773	*Fbxo5*	−1.44	8.43E-07	Proliferation
ENSMUSG00000022033	*Pbk*	−1.14	9.89E-07	Cell cycle
ENSMUSG00000026683	*Nuf2*	−1.13	2.20E-06	Mitosis
ENSMUSG00000027306	*Nusap1*	−1.25	2.96E-06	Mitosis
ENSMUSG00000035455	*Fignl1*	−1.25	5.25E-06	DNA repair
ENSMUSG00000035683	*Melk*	−1.37	6.40E-06	Cell cycle
ENSMUSG00000034311	*Kif4*	−1.09	1.30E-05	Mitosis
ENSMUSG00000046179	*E2f8*	−1.42	1.30E-05	Cell cycle
ENSMUSG00000020914	*Top2a*	−1.25	1.67E-05	Proliferation
ENSMUSG00000048922	*Cdca2*	−1.17	2.81E-05	Mitosis
ENSMUSG00000023940	*Sgo1*	−1.35	3.67E-05	Mitosis
ENSMUSG00000029910	*Mad2l1*	−1.05	3.67E-05	Mitosis
ENSMUSG00000032254	*Kif23*	−1.00	4.60E-05	Cytokinesis
ENSMUSG00000042489	*Clspn*	−1.24	9.07E-05	Cell cycle
ENSMUSG00000022034	*Esco2*	−2.04	1.10E-04	DNA replication
ENSMUSG00000036223	*Ska1*	−1.68	1.33E-04	Mitosis
ENSMUSG00000024989	*Cep55*	−1.12	1.44E-04	Mitosis & Cytokinesis
ENSMUSG00000025001	*Hells*	−1.26	3.15E-04	Proliferation
ENSMUSG00000048327	*Ckap2l*	−1.27	3.48E-04	Mitosis
ENSMUSG00000041498	*Kif14*	−1.16	4.06E-04	Proliferation
ENSMUSG00000017499	*Cdc6*	−1.34	6.07E-04	Cell cycle
ENSMUSG00000026669	*Mcm10*	−1.17	7.00E-04	DNA replication
ENSMUSG00000027379	*Bub1*	−1.37	8.14E-04	Mitosis
ENSMUSG00000032783	*Troap*	−1.01	8.56E-04	Proliferation
ENSMUSG00000007080	*Pole*	−1.03	9.90E-04	DNA repair
ENSMUSG00000022322	*Shcbp1*	−1.13	1.05E-03	Proliferation
ENSMUSG00000028212	*Ccne2*	−1.58	1.08E-03	Cell cycle
ENSMUSG00000031004	*Mki67*	−1.60	1.25E-03	Mitosis
ENSMUSG00000024795	*Kif20b*	−1.31	1.33E-03	Cytokinesis
ENSMUSG00000034329	*Brip1*	−1.33	1.46E-03	DNA repair
ENSMUSG00000037474	*Dtl*	−1.07	1.46E-03	DNA repair
ENSMUSG00000015880	*Ncapg*	−1.21	1.52E-03	Mitosis
ENSMUSG00000038379	*Ttk*	−1.35	1.62E-03	Mitosis
ENSMUSG00000047534	*Mis18bp1*	−1.51	1.88E-03	Mitosis
ENSMUSG00000039748	*Exo1*	−1.44	3.77E-03	DNA repair
ENSMUSG00000026196	*Bard1*	−1.01	3.88E-03	DNA repair
ENSMUSG00000046591	*Ticrr*	−1.13	5.52E-03	Cell cycle
ENSMUSG00000020897	*Aurkb*	−1.03	5.85E-03	Mitosis
ENSMUSG00000039396	*Neil3*	−1.46	5.85E-03	DNA replication
ENSMUSG00000028175	*Depdc1a*	−1.61	6.44E-03	Transcriptional regulation
ENSMUSG00000031629	*Cenpu*	−1.26	6.76E-03	Mitosis
ENSMUSG00000028718	*Stil*	−1.37	7.37E-03	Mitosis
ENSMUSG00000045328	*Cenpe*	−1.19	8.46E-03	Mitosis
ENSMUSG00000027326	*Knl1*	−1.57	1.09E-02	Mitosis
ENSMUSG00000034023	*Fancd2*	−1.30	1.13E-02	DNA repair
ENSMUSG00000021714	*Cenpk*	−1.12	1.25E-02	Mitosis
ENSMUSG00000036768	*Kif15*	−1.34	1.36E-02	Mitosis
ENSMUSG00000020330	*Hmmr*	−1.14	1.37E-02	Cell motility
ENSMUSG00000020493	*Prr11*	−1.18	1.51E-02	Cell cycle
ENSMUSG00000036777	*Anln*	−1.06	1.53E-02	Cytokinesis
ENSMUSG00000022360	*Atad2*	−1.24	1.65E-02	Proliferation
ENSMUSG00000027699	*Ect2*	−1.21	1.65E-02	Cytokinesis
ENSMUSG00000026605	*Cenpf*	−1.09	1.68E-02	Mitosis
ENSMUSG00000051235	*Gen1*	−1.44	2.14E-02	DNA repair
ENSMUSG00000017146	*Brca1*	−1.20	2.36E-02	DNA repair
ENSMUSG00000030528	*Blm*	−1.34	2.74E-02	DNA repair
ENSMUSG00000023919	*Cenpq*	−1.00	2.81E-02	Mitosis
ENSMUSG00000025758	*Plk4*	−1.11	2.87E-02	Cell cycle
ENSMUSG00000029414	*Kntc1*	−1.12	3.16E-02	Mitosis
ENSMUSG00000020185	*E2f7*	−1.26	3.72E-02	Cell cycle
ENSMUSG00000012443	*Kif11*	−1.31	4.19E-02	Mitosis
ENSMUSG00000031262	*Cenpi*	−1.07	4.19E-02	Mitosis

For heavy ion exposure, global gene expression profiling was not performed as the required four independent biological replicates for mRNA sequencing could not be collected during four independent beamtimes. Therefore, RT-qPCR experiments with RNA from single beamtimes were executed (Section 3.6.2). The replicates generated during one beamtime are not completely independent but they were derived from different astrocyte cultures, and they were irradiated separately.

#### 3.6.2. Selected target genes

As cell cycle control and proliferation genes were downregulated 24 h after exposure to 2 Gy X-rays in global gene expression profiling by RNA sequencing, a more detailed analysis of genes involved in cell cycle regulation after radiation exposure was performed (*Cdkn1a, Cdkn2a*). To consolidate the results obtained by immunofluorescence staining for GFAP expression and determine whether astrocyte reactivity was induced, *Gfap* mRNA levels were determined. Furthermore, the basal expression of IL-6 by astrocytes observed in the ELISA experiments led to a focus on genes involved in proliferation, inflammation and apoptosis (*Il1ß, Il6, Tnf* , *Tgfß1*).

**Figure 12:**
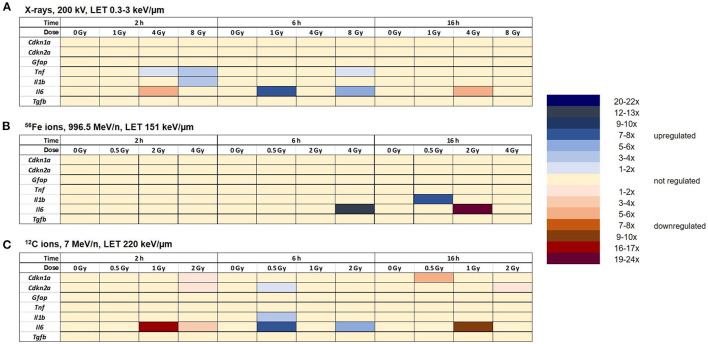
Radiation exposure of astrocytes discretely regulates the expression of some selected target genes without clear dose- and time-dependency. Expression of selected target genes in primary murine astrocytes at different time points after exposure to X-rays (*n* = 4) **(A)**, Fe ions (*n* = 4) **(B)** and C ions (*n* = 4) **(C)** was analyzed using RT-qPCR (*n* = 4) as described in Section 2.6.2. The samples were compared by one-way-ANOVA. Expression of significantly regulated target genes (*p* < 0.05) is shown by a color code for each gene, dose and time point.

Astrocytes were irradiated with different doses of X-rays (1 Gy, 4 Gy, 8 Gy), ^56^Fe ions (0.5 Gy, 2 Gy, 4 Gy) or ^12^C ions (0.5 Gy, 1 Gy, 2 Gy) and lysed 2 h, 6 h and 16 h afterwards. The relative changes in mRNA levels were determined and normalized to the expression level of *Hprt-1*. The results are summarized in Figure 12, detailed graphs are available in Supplementary material.

The expression of the cell cycle-associated genes *Cdkn1a* and *Cdkn2a* did not change significantly after X-irradiation (Figure 12A). The expression of *Gfap* was also not significantly regulated after exposure to X-rays. The expression levels of the inflammation-associated genes *Il1*β, *Il6*, and *Tnf* were upregulated 2 h and/or 6 h after irradiation with X-rays (*Il1*β*:* 2 h 8 Gy; *Tnf* : 2 h 4 Gy and 8 Gy; 6 h: 8 Gy). *Il6* was transiently upregulated 6 h after irradiation with 1 and 8 Gy X-rays, while in the samples exposed to 4 Gy, downregulation prevailed. Furthermore, irradiation with X-rays did not significantly change the expression of *Tgf*β at 2 h, 6 h and 16 h after irradiation.

After irradiation with ^56^Fe ions (Figure 12B), the expression of the cell cycle-associated genes *Cdkn1a* and *Cdkn2a* was not significantly changed. The expression levels of the astrocyte reactivity marker *Gfap* and the cytokine *Tgfß1* were not differentially regulated. *Tnf* expression varied strongly between the biological replicates, resulting in no significant changes. Also, expression of the inflammation-associated genes *Il6* and *Il1b* was not significantly altered except for an upregulation of *Il6* expression 6 h after exposure to 4 Gy Fe ions, followed by a downregulation 16 h after exposure to 2 Gy Fe ions, and an upregulation 16 h after exposure to 0.5 Gy Fe ions.

After irradiation with ^12^C ions (7 MeV/n), the expression of the cell cycle-associated genes *Cdkn1a* and *Cdkn2a* was not significantly changed except for a transient downregulation at 2 h after 2 Gy C ion irradiation (both) and 16 h after 0.5 Gy C ions (*Cdkn1a*) or 2 Gy C ions (*Cdkn2a*) (Figure 12C). The expression level of *Gfap, Tnf* , and *Tgfß1* did not change significantly after exposure to C ions. *Il1b* was transiently upregulated 6 h after exposure to 0.5 Gy C ions. The expression of the inflammation-associated gene *Il6* was upregulated at the 6 h time point (0.5 and 2 Gy). Additionally, a downregulation was observed 2 h and 16 h post-irradiation with 1 and 2 Gy and 16 h after irradiation with 1 Gy C ions.

## 4. Discussion

The radiation response of astrocytes is an important piece of the puzzle in understanding the effects of different ionizing radiation qualities on the brain as they represent the predominant cell type in the mammalian brain (22) and are crucial to normal brain function (83). Although the isolation of primary astrocytes is laborious and the results are sometimes variable, primary astrocytes from rodent embryos are recognized as a potent tool to study astrocytes, biology and mechanistic features (66, 84). As alternative cell models such as immortalized astrocytes and C6 glioma cells showed major differences in morphology, protein expression and functionality in comparison to primary astrocytes (84), we chose primary murine astrocytes for our studies. Overall, the radiation response of primary murine astrocytes was unremarkable with mostly minor changes for some doses and time points after exposure.

For coping with radiation-induced DNA damage, DNA repair is crucial. Here, it was shown that primary murine astrocytes repair DNA DSBs induced by X-rays with a kinetics that is comparable to other repair-proficient cell types (85–87). Fully functioning DNA repair was also suggested by Schneider et al. after investigation of X-rays-induced γH2AX foci in astrocytes that were differentiated from murine embryonic stem cell-derived neural stem cells (22). Also normal human astrocytes were capable to repair DNA DSBs induced by 10 Gy X-rays, and they even upregulated the expression of key proteins involved in non-homologous end joining (Ku70) and homologous recombination (RAD51) after irradiation (88) and they increased DNA repair when they were in a reactive state (89). For the radiation qualities with an LET of ~150 keV/μm, the number of 53BP1 foci was lower than the maximal number of γH2AX foci. This is in line with findings in murine neural stem/progenitor cells after γ-irradiation, in which γH2AX and 53BP1 did not fully colocalize and the number of 53BP1 foci was lower than the number of γH2AX foci in the same cell nucleus (90). After phosphorylation of H2AX, 53BP1 is recruited to the DNA DSB and it is involved in the DNA DSB repair pathway choice by controlling resection at the free DNA ends and thereby interfering with homologous recombination (91, 92). In retinal pigment epithelial RPE1 cells, 53BP1 was predominantly recruited to repair foci of cells in G0/G1 phase which was explained by its role in non-homologous end-joining, the DSB repair pathway available in mammalian cells during this cell cycle phase, while the 53BP1 foci were smaller during S phase (93). In G2 phase, 53BP1 foci might disappear faster than the γH2AX foci (94) and in general, the dynamics of radiation-induced γH2AX and 53BP1 foci disappearance can differ (95, 96). This might explain the lower number of 53BP1 foci compared to γH2AX foci that was observed in primary murine astrocytes in this work.

Simulated microgravity did not modulate γH2AX foci formation after exposure to X-rays and the subsequent repair of DNA DSBs by primary murine astrocytes. The question whether microgravity modulates the DNA damage response and more specifically, DNA repair, was addressed in several space experiments (97–100) and ground-based studies (52, 101) and they led to conflicting results. A growth-stimulating effect of microgravity and changes in gene expression might be contributors to microgravity effects on the DNA damage response, while DNA repair itself was mostly unaffected (52), which is in line with the results of this study. As a clear dose-dependent radiation response of astrocytes was observed only for DNA DSB induction and repair, simulated microgravity was incorporated in these experiments only and not extended to other biological endpoints investigated in this work.

The number of γH2AX foci that were induced by the same energy dose of iron ions was lower compared to X-rays. This finding is expected for high-LET radiation, as ionization occurs along tracks, resulting in a lower number of foci, which can contain more complex DNA damage and/or several DNA DSBs (85, 102). With higher LET, a lower number of average hits (and thereby, γH2AX foci) was expected after heavy ion exposure (Table 3). Furthermore, after ^56^Fe ion exposure, γH2AX foci formation and removal was delayed when compared to X-rays. This delayed repair is generally explained by the complexity of the heavy ion-induced DNA damage, requiring the coordination of several repair pathways (103).

To gain time for this DNA repair, cell cycle can be arrested at different checkpoints after ionizing radiation exposure. In murine astrocytes, no significant cell cycle changes were observed after exposure to X-rays. A clear G2 arrest which is usually induced in strongly proliferating mammalian cells after ionizing radiation exposure was not observed. Due to the low proliferation rate of primary astrocytes (a cell population doubling occurred after ~180 h in passage 1), accumulation in G2 phase may be negligible or completely absent. Gene expression profiling indicated downregulation of proliferation, cell cycle and mitosis genes. In RT-qPCR of selected target genes, no significant effects on the cell cycle regulation genes *Cdkn1a* (encoding p21^WAF/CIP1^) and *Cdkn2a* (encoding p16) which are involved in cell cycle arrests after ionizing radiation exposure and in senescence induction were observed after X-rays and Fe ions exposure. This is in line with the absence of a G2 arrest. Relative quantification of mRNA levels in RT-qPCR might obscure already high gene expression levels of *Cdkn1a* and *Cdkn2a* as indicators of senescence, but the presence of S-phase and G2-phase cells under all treatment conditions does not suggest a complete G1 cell cycle arrest in the primary murine astrocytes investigated in this work. A general downregulation of the DNA damage response signaling in astrocytes was described previously (22) additionally attributing to the absence of radiation-induced cell cycle arrest. The baseline cell cycle distribution in mock-irradiated cells was as follows: 40–50 % of cells were in G0/G1 and 50–60 % in S or G2 phase. For each experiment, new astrocytes were isolated and some variation for different isolates might be attributed to such isolate batch effects and to different timelines in the preparation of the beamtimes at the heavy ion accelerator. Therefore, mock-irradiated controls were generated for each experiment to account for such batch variations. The cells in G0/G1 might reside in a quiescent state (G0) that permits subsequent cell division upon stimulation (e.g., withdrawal and re-addition of FBS) or in G1 as part of the actively cycling cells (104).

Astrocyte reactivity (105) was evaluated by proliferation, GFAP and cytokine expression. Proliferation of astrocytes usually increases upon reactivity, inducing cell infiltration to damage sites in the CNS (23). As Ki-67 is the most cited proliferation marker that can be determined by immunofluorescence staining with highest levels during G2 phase and mitosis (106, 107), the fraction of Ki-67 positive cells after exposure of astrocytes to X-rays was quantified and found to be largely unaffected by X-irradiation at around 10 %. A similar low percentage of proliferating cells of 10–25 % was also observed in primary rat astrocytes (66) and in adult mouse astrocytes (108). As the proliferation rate decreased in higher passages (108), only passage 1 astrocytes were used in this work. A higher proliferation of murine astrocytes could be achieved by adding 20 % FBS instead of 10 % (108). Also, in presence of TNF-α, the percentage of Ki-67^+^ cells increased to 25–30 %, although this effect was not significant due to large standard errors. Increased astrocyte proliferation in response to the cytokines TNF-α and IL-1β increase was described previously, supporting the results of this work (79). As X-ray doses up to 8 Gy did not significantly change Ki-67 expression, this marker was not used in the heavy ion experiments.

In this work, astrocytes basally expressed GFAP without significant changes after exposure to low–LET radiation up to a dose of 8 Gy. In the context of research to improve radiotherapy of brain tumors, reactive gliosis after irradiation with higher doses (10 Gy) and in systemic context (whole-body X-irradiation of mice) was observed based on upregulation of GFAP in the brain (109). Increased GFAP expression was also documented 6 h and 24 h after head-only X-irradiation (15 Gy) of rats (15) and of mice (20 Gy) (110). As absence of serum response factor (SRF) resulted in increased GFAP expression in SRF knockout mice, the isolated culture of primary astrocytes in presence of serum-containing medium could be an explanation for suppressed GFAP expression and absence of increase after irradiation (111). While isolated culture of astrocytes offers the advantage that the specific radiation response of this cell type can be analyzed by methods not suitable for co-cultures or brain slices, responses that occur only in the multicellular context of brain tissue cannot be addressed. Here, it has to be considered that astrocytes' responses to CNS insults are a multicellular process in which the reaction of all cell types in the brain including microglia, oligodendrocytes or neurons and their release of signaling molecules is integrated to activate astrocytes (23). For increased proliferation of astrocytes in response to injury, crosstalk of astrocytes with macrophages is required (112). In cultured rat brain astrocytes incubated with media supernatants from X-irradiated (2 and 10 Gy) microglia, GFAP expression was increased after 24 h (15), indicating an important role of microglia in astrogliosis induction by ionizing radiation exposure. Also, in co-culture with endothelial cells in an organ-on-a-chip-model, exposure to 0.3 Gy and 0.82 Gy ^56^Fe ions (600 MeV/n, LET 170 keV/μm) increased GFAP expression in astrocytes 3 days after exposure (113).

Immunostaining of p65 and IL-6 ELISA revealed that in primary murine astrocytes, basal activity of the NF-κB pathway was present resulting in continuous IL-6 expression and secretion which was not further enhanced by exposure to X-rays or C ions.

The absence of radiated-induced NF-κB activation correlates well with the absence of radiation-induced GFAP expression as GFAP expression is regulated by NF-κB by a κB binding site in the GFAP promoter region (114). In this context, the previously observed downregulation of DNA damage response signaling including ATM activity in astrocytes (22) is of interest as ATM is a key player in ionizing radiation-induced NF-κB pathway activation (70), possibly explaining the absence of NF-κB activation by the radiation qualities investigated in this work.

In the CNS, the proinflammatory cytokine IL-6 is predominantly produced by astrocytes and is involved in cell-cell communication and reactivity of astrocytes. IL-6 could exert some autocrine actions in astrocytes (74, 115, 116). Both, proliferative (117) and antiproliferative actions (118) of IL-6 in astrocytes have been described. Transient local cytokine secretion might promote the brain's recovery after injury, but long-term upregulation might result in damage (76). IL-6 expression is expected to be found in a senescence-associated secretory profile (SASP), a phenotypic shift leading to premature or stress-induced cellular senescence (119–121). An irreversible cell cycle arrest is a major characteristic of cellular senescence (49, 50). In human fibroblasts, cell populations with < 10 % proliferative cells were designated as non-dividing senescent cultures (51). Here, no significant reductions in Ki-67^+^ astrocytes were observed. Therefore, this finding of basal IL-6 secretion suggests that these cells quickly adopt an only partially senescent phenotype in isolated culture, which is not further enhanced by *in vitro* exposure to ionizing radiation. TP53 was reported to regulate cellular senescence in astrocytes induced by ionizing radiation exposure (119). The absence of a decided TP53 gene expression signature (including e.g., the expression of *Cdkn1a*—p21) in the RNA sequencing data after X-rays exposure might explain why the radiation response of primary astrocytes was so reluctant. While TP53 in astrocytes was related to various disease processes (122), its role in the DNA damage response of astrocytes remains elusive. In astrocytes that were differentiated from murine embryonic stem cell-derived neural stem cells and exposed to 10 Gy or even 50 Gy X-rays, no TP53 activation occurred 1 h and 24 h after irradiation, and the expression levels of the TP53 target genes GADD45a, BAX and PUMA remained largely unchanged, only CDKN1A expression was upregulated (22). This absence of a TP53-mediated transcriptional response after exposure to X-rays was also observed in cortical astroglia cell cultures from 1-d old mouse pups (123) and might be an important factor for the observed radioresistance of astrocytes (22). However, in activated proliferating astrocytes, exposure to 4 Gy X-rays activated TP53 (124), indicating that the reactivity status might influence the TP53 response of astrocytes.

Another possibility is that a senescent phenotype is present because of high basal expression of *Cdkn1a* and *Cdkn2a*, the major mediators of senescence-associated proliferation arrest (121), that did not further increase after treatment. *In vivo*, cellular senescence describes a state in which astrocytes do not replicate but remain alive in the tissue, producing pro-inflammatory and neurotoxic factors, and contributing to CNS damage (119, 125). Such aging of astrocytes was associated with a smaller pool of synaptic vesicles in co-cultured neurons and decreased neuroprotective capacity (126, 127).

The data acquired in this work provide an indication that exposure of primary murine astrocytes in isolated culture to X-rays or heavy ions did not result in astrocyte reactivity. Therefore, further experiments were not performed to characterize reactivity in more detail by including various markers (vimentin, leucine zipper kinase, and nestin) (23, 111, 128) and pathways involved in reactivity such as STAT3, cyclic adenosine monophosphate (cAMP) or C-Jun-N-terminal kinase (JNK) (23, 129).

Sequencing of mRNA isolated from X-irradiated murine astrocytes revealed that a low dose of 0.1 Gy had no effect on global gene expression 6 and 24 h after irradiation. Gene expression changes were observed only at the late time point 24 h after irradiation, and for the higher dose of 2 Gy. The number of affected genes was low, the majority being downregulated. This downregulation was moderate and affected mostly genes involved in proliferation and DNA repair. As both processes were not significantly affected after irradiation of astrocytes, the biological role of these downregulations remains unclear. The downregulation was not observed at the 6 h time point while DNA DSB repair was still ongoing. The majority of the nine downregulated DNA repair genes is involved in homologous recombination (*Fignl1, Brip1, Fancd2, Gen1, Brca1, Blm*) which is not expected to be the prominent DNA DSB repair pathway in astrocytes. Therefore, at the current stage, no strong indications of a high functional relevance of the small reduction in expression of DNA repair genes can be derived. No overlap of the profiles was observed when comparing this gene expression profile to the expression profile of reactive astrocytes in two mouse brain injury models, in which several hundreds of genes were upregulated (105). This suggests that the expression profile observed in this work is not indicative of reactive astrogliosis, although it has to be considered that the gene expression profile of reactive astrocytes was described to be specific for a given injury (105).

Only two genes were upregulated in response to 2 Gy X-rays: *Svop* and *Abhd11os*. The synaptic vesicle protein SVOP is described as a 548-aa protein of ~60 kDa (130) with 12 transmembrane regions (131) capable of binding nucleotides [e.g., nicotinamide adenine dinucleotide (NAD)] (132) and of transporting nicotinate (133). Its expression was described to be limited to the CNS (134); in the adult mouse brain it was predominantly found in hippocampus and cerebellum (130). Based on its structure as a transporter-like protein (135) and functional studies performed so far, a possible role in synaptic vesicle uptake/transport is assumed that is not required for survival under normal conditions (136). In humans, abnormal methylation of the SVOP gene located on chromosome 12 (137) was correlated with prognosis of glioblastoma (138). Not much is known on the function of long non-coding RNA (lncRNA) *Abhd11os* (139) in astrocytes. The human homolog ABHD11 antisense RNA 1 (ABHD11-AS1) was found to be highly expressed in gastric, lung, breast, colorectal, thyroid, pancreas, ovary, endometrium, cervix, and bladder cancer and was therefore suggested as biomarker for diagnosis and prognosis (140). In mouse models of Huntington's disease, expression of *Abhd11os* was reduced (139) or dysregulated (141) and its overexpression had neuroprotective effects in mice against mutant huntingtin-induced toxicity (139). Besides these findings in cancer and Huntington's disease models, the role of *Abhd11os* expression in myocardial infarction was addressed: Increased *Abhd11os* expression was found in a rat myocardial ischemia/reperfusion injury model and hypoxia/reoxygenation-treated cardiomyocytes (142). This upregulation of *Abhd11os* inhibited proliferation of cardiomyocytes but promoted cell apoptosis, while downregulation of *Abhd11os* inhibited apoptosis of cardiomyocytes thereby attenuating the injury (142). Interestingly, after whole-body irradiation of mice, the expression of the *Abhd11os* increased dose-dependently in heart tissue (143), in line with the X-rays-induced upregulation observed in murine astrocytes in this study.

To assess whether gene expression might be more modulated after exposure to higher doses or other radiation qualities (Fe and C ions), or only transiently affected, several genes of interest were analyzed by RT-qPCR: *Cdkn1a* and *Cdkn2a*, that are involved in cell cycle progression *Gfap* as marker for astrocyte reactivity; the cytokines *Tnf* , *Il1*β and *Il6*, being involved in inflammation, proliferation and apoptosis; *Tgf*β*1*, which acts in anti-inflammatory and anti-apoptotic manner. For these genes, no clear dose dependence of up- or downregulation was observed, and some regulations were transient. For example, X-irradiation with 8 Gy caused only a transient upregulation of *Il6* at the time point 6 h.

Due to the limited availability of beamtimes at heavy ion accelerators, the heavy ion experiments could not be repeated in independent beamtimes, but biological replicates isolated from different animals were included and the sample size of each biological replicate contained several thousand cells. Batch effects of different isolates were observed for example in terms of the higher basal IL-6 secretion in the C ion (7 MeV/n) experiments at GSI compared to the experiments with X-rays at DLR. Furthermore, not all biological endpoints could be analyzed for all four radiation qualities due to beamtime time restrictions. Nonetheless, the results of the heavy ion experiments were interpreted only as possible trends if statistical tests were not possible. Also, due to the low energy of the carbon ions, the UNILAC beamtime required a different experimental setting where astrocytes were seeded in petri dishes and kept in a reservoir with cell culture medium for irradiation.

The choice of radiation doses used in this work was based on the average mission doses on ISS [6 months ~ 90–150 mSv (144, 145)] and a 1,000-days Mars mission [~340–1,000 mSv, depending on solar activity and shielding (146)]. Higher doses up to 8 Gy were added to generate dose response curves. As the relative biological effectiveness (RBE) for the investigated biological endpoints in astrocytes is not known, we used mostly the same dose range for X-rays and heavy ions to generate data from which the RBE could be derived. As in most of the space radiobiological *in vitro* experiments, the effects of an acute radiation exposure were investigated—a protracted radiation exposure of cultured cells with heavy ions over 6 months to 3 years, imitating the mission durations, is simply not feasible. The extrapolation of the effects of this acute high-dose rate exposure to chronic low-dose rate exposure requires some assumptions that are usually considered in terms of a dose- and dose-rate reduction factor (DDREF) (147–150), which, in worst case where a lower dose rate does not alleviate the damage, is 1.

In conclusion, primary murine astrocytes were shown to be fully repair-proficient for DNA DSBs induced by low- and high-LET radiation. They seemed to be quite radioresistant and a comprehensive DNA damage response also including cell cycle arrests and cell death was absent. In isolated culture, they did not shift toward astrocyte reactivity but the indicators of a senescent phenotype warrant further investigation. Also, based on the findings of this work, it seems that the question of astrogliosis or astrosenescence cannot be answered when astrocytes are isolated from their natural microenvironment in the brain. More complex systems such as co-cultures, multicellular culture models (59), organ-on-a-chip models (113), brain slices or brain organoids could be interesting models to study astrocytes' response to space-relevant radiation qualities embedded in the cellular crosstalk within the brain.

## Data availability statement

The RNA Seq datasets presented in this study can be found in online repositories. The name of the repository and accession number is NCBI Gene Expression Omnibus (GEO) GSE215383 - Astrocytes' Radiation Response to be found under the link https://www.ncbi.nlm.nih.gov/geo/query/acc.cgi?acc=GSE215383.

## Ethics statement

The animal study was reviewed and approved by Landesamt für Natur, Umwelt und Verbraucherschutz Nordrhein-Westfalen, LANUV, Germany on 4 December, 2017, Nr. 84-02.04.2017.A319.

## Author contributions

Conceptualization and writing—original draft: CEH, JK, and MDR. Data curation: MDR, SH, and EW. Formal analysis and visualization: CEH, MDR, SH, EW, and JK. Funding acquisition: CEH and SD. Investigation: CEH, BK, MDR, SH, EW, JK, SD, and HN. Methodology: CEH, MDR, SH, EW, JK, and SD. Project administration: CEH and JK. Resources: CEH. Supervision: CEH, JK, SD, and CL. Writing- review and editing: HN, CEH, MDR, BK, SH, and CL. All authors have read, agreed to the published version of the manuscript, approved the submitted version, agree to be personally accountable for the author's own contributions and for ensuring that questions related to the accuracy or integrity of any part of the work, even ones in which the author was not personally involved, are appropriately investigated, resolved, and documented in the literature.
